# Thermal and Hydrodynamic Environments Mediate Individual and Aggregative Feeding of a Functionally Important Omnivore in Reef Communities

**DOI:** 10.1371/journal.pone.0118583

**Published:** 2015-03-16

**Authors:** Desta L. Frey, Patrick Gagnon

**Affiliations:** Department of Ocean Sciences, Ocean Sciences Centre, Memorial University of Newfoundland, St. John’s, Newfoundland and Labrador, Canada, A1C 5S7; University of Connecticut, UNITED STATES

## Abstract

In eastern Canada, the destruction of kelp beds by dense aggregations (fronts) of the omnivorous green sea urchin, *Strongylocentrotus droebachiensis*, is a key determinant of the structure and dynamics of shallow reef communities. Recent studies suggest that hydrodynamic forces, but not sea temperature, determine the strength of urchin-kelp interactions, which deviates from the tenets of the metabolic theory of ecology (MTE). We tested the hypothesis that water temperature can predict short-term kelp bed destruction by *S*. *droebachiensis* in calm hydrodynamic environments. Specifically, we experimentally determined relationships among water temperature, body size, and individual feeding in the absence of waves, as well as among wave velocity, season, and aggregative feeding. We quantified variation in kelp-bed boundary dynamics, sea temperature, and wave height over three months at one subtidal site in Newfoundland to test the validity of thermal tipping ranges and regression equations derived from laboratory results. Consistent with the MTE, individual feeding during early summer (June-July) obeyed a non-linear, size- and temperature-dependent relationship: feeding in large urchins was consistently highest and positively correlated with temperature <12°C and dropped within and above the 12–15°C tipping range. This relationship was more apparent in large than small urchins. Observed and expected rates of kelp loss based on sea temperature and urchin density and size structure at the front were highly correlated and differed by one order of magnitude. The present study speaks to the importance of considering body size and natural variation in sea temperature in studies of urchin-kelp interactions. It provides the first compelling evidence that sea temperature, and not only hydrodynamic forces, can predict kelp bed destruction by urchin fronts in shallow reef communities. Studying urchin-seaweed-predator interactions within the conceptual foundations of the MTE holds high potential for improving capacity to predict and manage shifts in marine food web structure and productivity.

## INTRODUCTION

Shallow reef communities in high-latitude seasonal seas are exposed to considerable variation in thermal and hydrodynamic environments [1–3]. Mobile consumers in these environments typically exhibit behavioral shifts across gradients of water temperature and wave action to balance physiological requirements and biomechanical limitations [4–6]. Alterations to species interactions ensuing from behavioral shifts can ultimately shape population dynamics and biodiversity patterns [7–9]. A number of studies show that displacement toward, and consumption of, prey in mobile solitary invertebrates are respectively positively and negatively related to water temperature and wave action [10–12]. Yet, we know much less about how temperature and wave action affect foraging in mobile gregarious invertebrates [13–15]. Understanding plasticity in foraging and interspecific interactions of functionally important consumers is a key step toward anticipating and mitigating alterations to reef communities resulting from ongoing global shifts in sea temperature and state [16–19].

Because of its high destructive potential, the omnivorous green sea urchin, *Strongylocentrotus droebachiensis*, has become one of the most scrutinized organisms in studies of subtidal community dynamics in the northern hemisphere [20]. In eastern Canada, the destruction of foundational [21] kelp beds by dense green sea urchin aggregations (fronts) is a key determinant of the structure and dynamics of shallow reef communities [22–27]. In Nova Scotia and the northern Gulf of St. Lawrence, fronts can destroy kelp beds and associated biota at rates as high as 4 m month^-1^, leaving behind pavements of grazing-resistant, red coralline seaweeds termed “barrens” [23, 24, 28]. Recent studies of relationships among urchin front formation, kelp bed destruction by fronts, and environmental variability in Nova Scotia suggest that wave action has a much greater effect than sea temperature on the regulation of urchin-kelp interactions across the 0–18°C range. For example, urchin density at fronts has been negatively correlated with wave height, with no detectable effect of water temperature on the rate of advance of, and urchin density at, fronts below a suggested threshold of ~17°C [28, 29]. Feehan et al. [30] propose that the lack of a density threshold for destructive grazing in pre-existing gaps in kelp canopies (which is inconsistent with other studies in eastern Canada [23–25, 29]) was due to insufficient wave action allowing urchins to aggregate and feed upon kelp more readily. Temperature in the latter study also did not appear to explain any of the observed variation in urchin-kelp dynamics [30].

The notion that temperature has virtually no effect on these relationships challenges the tenets of the metabolic theory of ecology (MTE), which links the performance of individual organisms to the ecology of populations, communities, and ecosystems [31]. According to the MTE, individual performance, and hence species interactions, is largely determined by 1) temperature, which affects biochemical reactions; and 2) body size, which affects the minimal rate of energy expenditure necessary for survival [31, 32]. In general, rates of biochemical reactions increase with both temperature and body size, so long as temperature is within the range of normal activity, which for most organisms lies between 0 and 40°C [31]. This, in theory, makes ectothermic organisms such as urchins particularly sensitive to variations in the thermal environment. We argue that the apparent lack of a relationship between sea temperature and rates of individual and aggregative feeding in *S*. *droebachiensis*, may be because: 1) most of the studies yielding this conclusion are observational, which does not allow for proper testing and partitioning of causal links between temperature, wave action, and feeding; 2) wave conditions over which urchin-kelp interactions were measured were generally too high for temperature to emerge as a significant factor; and 3) effects of temperature on individual and aggregative urchin performance (in this case displacement and feeding), and how they may change temporally, have been largely overlooked.

In the present study, we integrate experimental, observational, and analytical approaches to test the hypothesis that water temperature can predict short-term (over a few months) kelp bed destruction by *S*. *droebachiensis* in calm hydrodynamic environments. This hypothesis stems from the argument that under low hydrodynamic forces, urchin displacement, and hence the capacity to aggregate at the lower margin of kelp beds and consume kelp, should increase proportionally with temperature. It assumes that short-term changes in density at the front result primarily from increased immigration or emigration of urchins from or to the adjacent barrens and kelp bed, as opposed to broader-scale processes such as mortality or the recruitment of new individuals from reproductive events. Specifically, we carry out two laboratory experiments to investigate effects of water temperature and urchin body size on individual feeding, as well as of wave action on aggregative feeding at two times of year. We quantify variation in kelp-bed boundary dynamics, sea temperature, and wave height over three months at one subtidal site in Newfoundland to study relationships between environmental variability and urchin density at the kelp-barrens interface. We use results from the latter survey to generate data against which we test the validity of thermal tipping ranges and regression equations derived from laboratory results. We provide the first compelling evidence that sea temperature, and not only hydrodynamic forces, can predict kelp bed destruction by urchin fronts in shallow reef communities.

## MATERIALS AND METHODS

### Study and collection sites

The present study was conducted with *Strongylocentrotus droebachiensis* and *Alaria esculenta* at, or collected from, two adjacent sites on the north shore of Bay Bulls, Newfoundland (Canada): Bread and Cheese Cove (BCC, 47°18'30.8'' N, 52°47'19.1'' W) and Cape Boone Cove (CBC, 47°18'30.4'' N, 52°47'11.1'' W). All necessary permits for sampling and collecting seaweeds and urchins (see below) were obtained prior to sampling and experimentation in accordance with the Canadian Council of Animal Care guidelines. No specific locational permits were required for seaweed and urchin sampling and collection in Bay Bulls, and no threatened or endangered species were at risk of incidental capture. The BCC and CBC sites are separated by a rocky outcrop, Bread and Cheese Point, which extends ~150 m into the bay along a north-south axis. The seabed at both sites is composed of gently sloping bedrock, to a depth of ~15 m (chart datum), with scattered boulders between 3 and 5 m at BCC. At BCC, kelp beds, mainly *A*. *esculenta* and *Laminaria digitata*, dominate the 0–2 m depth range, followed by an extensive urchin (*S*. *droebachiensis*) barrens to a depth of ~15 m. Transient beds of the annual, acidic, brown seaweed *Desmarestia viridis* establish every year in this barrens [33] and intersperse with a few stands of the grazing-resistant kelp *Agarum clathratum* [34]. At CBC, an extensive (several 100s of m^2^) kelp bed dominated by *A*. *esculenta* establishes to a depth of ~9 m during spring, followed in deeper water by an urchin barrens. Scattered patches of *L*. *digitata* develop in the *A*. *esculenta* bed between 0 and 4 m.

### Collection and acclimation of urchins prior to experimentation

Urchins used in the two laboratory experiments described below were hand collected by divers at depths of 3 to 6 m at BCC between 18 April and 27 September, 2012. They were transported in large containers filled with seawater to the Ocean Sciences Centre (OSC) of Memorial University of Newfoundland. Upon arrival at the OSC (<5 hours after collection), urchins were transferred to 330-L holding tanks supplied with ambient flow-through seawater pumped in from a depth of ~5 m in the adjacent embayment, Logy Bay. Each holding tank contained one group of 200 urchins fed every two days with 25 g (wet weight) of freshly collected blades of *Alaria esculenta* cut into pieces of ~2.5 x 2.5 cm (in the present study all organisms were weighed with the same balance with a precision of ±0.1 g; PB3002-S/FACT; Mettler Toledo). Urchin feces and unconsumed kelp, if any, were removed from the holding tanks prior to adding new kelp. Urchins were used in the experiments within 1–2 weeks after collection.

### Experiment 1: water temperature, body size, and individual urchin feeding

To investigate effects of water temperature and body size on individual feeding, we used a factorial experiment, Experiment 1, in which small, 25–35 mm in test diameter (t.d.), and large, 45–60 mm t.d., urchins were allowed to graze *Alaria esculenta* in seawater at six temperatures: 3, 6, 9, 12, 15, and 18°C. Our objective was to examine individual feeding during the first few weeks of summer, when urchin aggregation and grazing increase at the lower margin of kelp beds in eastern Canada [23, 24, 28]. We chose these temperature treatments because sea temperature in coastal Newfoundland, including at BCC, generally increases by 10–15°C between June (~1–2°C) and early August (~12–16°C) [35, 36].

We ran the experiment from 22 June to 28 July, 2012. Trials lasted 22 h (preliminary trials showed demonstrable kelp consumption over this period), and were conducted in three adjacent water baths (GD120L; Grant). The volume of each bath enabled running simultaneously one replicate of six of the 12 experimental treatments. Each full run was therefore completed over two consecutive days by applying three randomly chosen temperature treatments on the first day and the remaining three temperatures on the second day. Temperature treatments were assigned randomly to each bath on each day. On each day, one group of three small urchins and one group of three large urchins were each introduced to one of two 5-L plastic containers in each bath pre-filled with seawater from the holding tanks. Mean daily water temperature at BCC and in the holding tanks supplied from Logy Bay varied simultaneously from 4.2°C to 12.4°C, meaning that urchins were exposed to the same thermal conditions as in their natural habitat prior to being used in the experiment. As a result, urchins in ~12% of the trials were exposed to changes in temperature of up to 10–13°C. Changes of this magnitude may qualify as a shock. However, they do occur in coastal Newfoundland (including BCC) in early summer, with relatively frequent drops and rises of up to 10°C over the course of only a few hours to days [35, 36]. We did not acclimate the urchins to the experimental temperature treatments because 1) incorporating the natural thermal history of urchins into trials was a more accurate representation of natural processes affecting individual urchin feeding over the short term; and 2) the variable thermal environment to which they were exposed prior to trials made it impossible to determine a proper acclimation time for each temperature treatment. Nevertheless, the water in each container was gradually cooled or warmed to the desired experimental temperature over the four hours preceding the onset of all trials to facilitate the thermal transition of urchins.

Each trial began with the introduction, in each bath, of 10±0.5 g (wet weight) of freshly collected *A*. *esculenta* blades cut in ~2.5 x 2.5 cm pieces to each of the two containers with urchins and a third container with no urchins. Containers with kelp but no urchins were used to correct kelp tissue loss to grazing for autogenic loss or gain. The order of the three containers in each bath was determined randomly. The unconsumed kelp was wet weighed at the end of trials. We used the equation: Kelp loss = [(*T*_o_ × *C*_f_ / *C*_o_)—*T*_f_] to obtain the corrected kelp loss in each container with urchins, where *T*_o_ and *T*_f_ are the initial and final weights of kelp tissues exposed to urchins, respectively, and *C*_o_ and *C*_f_ are the initial and final weights of the corresponding autogenic control, respectively [33].

We used feeding rates in each container to calculate the mean feeding rate for each temperature and urchin body size treatment. Feeding rate was obtained by dividing the corrected kelp tissue loss by the number of urchins (three) and duration of trial (22 h). Each trial was run with new urchins and kelp. A gentle stream of air bubbles was continuously injected in each container with aquaria pumps (Maxima, Hagen) to maintain oxygenation. The 12 experimental treatments were replicated eight times.

### Experiment 2: wave action, season, and aggregative urchin feeding

In the present study, “wave action” refers to the combined effects of hydrodynamic forces, which affect the ability of mobile consumers to move toward (direct effect) and contact (indirect effect) sweeping algal fronds. Urchins undergo both effects in natural habitats, which ultimately modulate foraging [37]. Accordingly, we studied the overall impact of wave action on feeding, rather than the direct and indirect effects separately.

**Fig 1:**
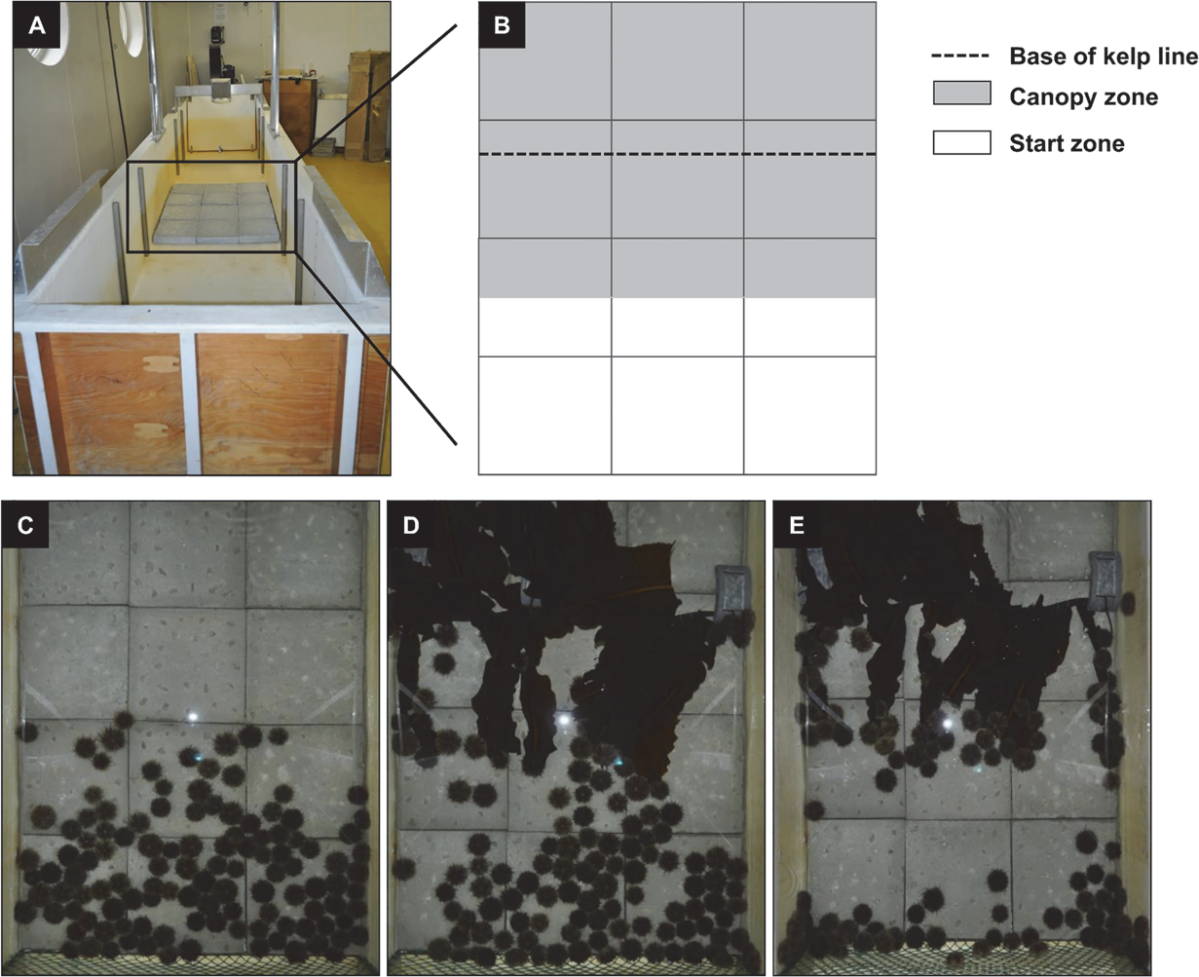
Oscillatory wave tank used in Experiment 2. (A) Position of the experimental area [3×4 grid of concrete tiles of 0.3×0.3×0.05 m each], and (B) relative positions of the kelp (*Alaria esculenta*) line, zone of maximum canopy cover [Canopy zone], and zone to which green sea urchins (*Strongylocentrotus droebachiensis*) were introduced prior to the onset of trials [Start zone]. The sequence at the bottom shows urchins at (C) t = 0 [prior to introducing the kelp line], (D) t = 1 h, and (E) t = 6 h [end] of a trial at a wave velocity of 0.1 m s^-1^ (see Materials and methods for details).

To investigate effects of wave action on aggregative feeding, we conducted a microcosm experiment, Experiment 2, in an oscillatory wave tank (Fig. 1), which simulated the wave-induced sweeping motion of kelp blades in natural habitats [38]. The experiment was conducted in spring 2012, and again in late summer 2012, to test the prediction that aggregative feeding is generally lower in spring than summer. Groups of 118 large (40–60 mm t.d.) urchins, corresponding to 292 individuals m^-2^ at the onset of trials, were allowed to graze *Alaria esculenta* sporophytes at four wave velocities: 0.0 m s^-1^ (Null), 0.1 m s^-1^ (Low), 0.2 m s^-1^ (Intermediate), and 0.3 m s^-1^ (High) (peak longitudinal velocity measured in the centre of, and at ~5 cm above, the surface of the experimental area with a Doppler current meter, Vector Current Meter; Nortek). Urchin density paralleled that at fronts at the lower edge of *A*. *esculenta* beds in the northern Gulf of St. Lawrence [24, 39]. Wave velocity included the threshold value of ~0.3 m s^-1^ above which displacement in *S*. *droebachiensis* virtually ceases [37]. Sporophytes formed a line similar to the lower edge of a kelp bed (see below). We used a fixed frequency of 15 wave cycles min^-1^ in treatments with waves because 1) we were interested in the effects of water velocity on aggregative feeding, rather than the effects of wave frequency per se; and 2) it reflects the general wave frequency under moderate winds at our collection and survey sites.

Trials were conducted on a 3 x 4 grid arrangement of concrete tiles (12 tiles, each 0.3 x 0.3 x 0.05 m), yielding an experimental area of 1.08 m^2^ (Fig. 1). The grid was located in the centre of the wave tank, where sinusoidal waves caused kelp blades to sweep back and forth at the onset of trials, when none of the urchins were in contact with the blades (Fig. 1). The grid was delimited longitudinally by the tank walls, and transversally by nylon netting with 2.5-cm mesh to restrict urchins to the experimental area. Preliminary measurements of water velocity with and without netting showed no perceptible changes along the *u*, *v*, and *w* vectors (in the *x*-, *y*-, and *z*-direction, respectively). The upper surface of the tiles was sculpted with holes, cracks, and waves to simulate natural bedrock heterogeneity. The kelp line consisted of *A*. *esculenta* sporophytes (40 cm in length), with stipes wedged into a split (at 1.5 cm intervals) made along a rubber hose (84 cm in length, 1.5 cm in diameter) and held in place by winding electrical tape around the hose. During trials, the hose was anchored down by 3-kg lead weights, and oriented so as to maintain kelp stipes at an angle of ~45° to the bottom of the tank, towards the urchins. In this position, the blade tips touched the bottom, mimicking what happens with kelp at the lower edge of the kelp fringe. We made several kelp lines, which we used in alternation from one trial to another. Between trials, the lines were maintained in holding tanks supplied with ambient flow-through seawater and new sporophytes were added to each line to replace those grazed during trials. The wet weight of the kelp line (including hose and tape) was determined before and after trials after shaking the line gently until no water came off. Kelp weight on the line was standardized at the onset of each trial by trimming a few sporophytes to a total line weight of 650 (±32.5) g.

Each trial lasted six hours to allow sufficient time for urchins to form fronts and consume detectable amounts of kelp at all wave velocities as determined with preliminary trials. All (118) urchins were introduced, oral surface down, to the “Start zone”, defined by the surface area (0.405 m^2^) of the outermost 1.5 rows of cross-current tiles in the grid (Fig. 1). Urchins were allowed to explore the experimental area in the absence of waves for one minute following the placement of the last individual. In trials with waves, the motor was turned on to create an initial wave velocity of 0.1 m s^-1^. The velocity was gradually increased over the next two and five minutes in the 0.2 and 0.3 m s^-1^ treatments, respectively, by adding water to the tank. This gradual increase was necessary to allow urchins in these two treatments to adapt to higher hydrodynamic forces and avoid dislodgement (in preliminary trials a few urchins detached from the tank bottom when velocity was increased more quickly). Urchins at all velocity treatments were allowed to move for a total of 6 min, with no change in velocity in the 0.0 and 0.1 m s^-1^ treatments. Waves were then stopped, and urchins that had left the start zone (generally <10 individuals in the 0.2 and 0.3 m s^-1^ treatments) were moved back into it to standardize the initial urchin distribution among trials. The onset of the trial (t = 0) was marked by the introduction of the kelp line to the other end of the grid, so as to have a space of ~40 cm between the kelp blades and nearest urchins. The motor was turned on again in treatments with waves. At the end of the trial (t = 6 h) we stopped waves (as required), counted and removed urchins that were consuming kelp, and removed the kelp line. We then photographed the experimental area with a digital camera (D5000; Nikon) located 1.3 m above the water surface, and weighed the kelp line. The tank was emptied, and feces and occasional kelp debris were removed. New seawater was added to the tank in the hour preceding each trial.

Kelp loss to feeding was corrected for autogenic loss or gain as determined from trials in which kelp lines were exposed for 2 h to the same velocity treatments as above (n = 5 [spring] and 3 [summer] for each wave treatment), except no urchins were introduced to the tank. Preliminary trials showed no difference in kelp loss or gain between 2-h and 6-h trials. The same equation as in Experiment 1 was used to determine the corrected kelp loss in each trial. We used feeding rates in each trial to calculate the mean feeding rate for each velocity treatment in each season. Feeding rate was obtained by dividing the corrected kelp tissue loss by the number of urchins (118) and duration of trial (6 h). We used direct counts, images of the experimental area at the end of trials, and PhotoImpact v6.0 (Ulead Systems, Inc.), to determine the numbers of urchins: 1) feeding on kelp; 2) underneath [and not feeding] the kelp canopy; 3) on the tiles, outside of the area swept by kelp; and 4) on the longitudinal walls of the tank and transverse nettings, collectively termed “the walls”. Urchins in the latter three categories respectively provided an indication of the tendency and ability of urchins to penetrate the kelp line at the lower margin of kelp beds, remain more or less stationary on a flat surface like in urchin barrens, and displace and take higher risks of dislodgement by climbing on vertical surfaces like rocks in barrens and kelp within beds.

Each wave velocity treatment was replicated eight times during spring (23 April to 30 May), and seven times during summer (26 August to 3 October), 2012. We blocked trials over time within each season by running one replicate of each treatment on four consecutive days (one trial per day). The order of the treatments among days was randomized in each block of four days. We alternated the position of the kelp line between the two transverse edges of the experimental area (and hence that of the start zone for urchins) between trials. The tiles were reshuffled randomly within the grid before the start of each trial. Each trial was run with new urchins. Water temperature in the wave tank during the spring and summer trials was 5.1 (±0.2)°C and 13.9 (±0.4)°C, respectively.

### Field observations: water temperature, wave action, and kelp-bed boundary dynamics

To test the hypothesis that water temperature can predict short-term kelp bed destruction by *Strongylocentrotus droebachiensis* in calm hydrodynamic environments, we studied changes over three months in: 1) the absolute position of the lower limit of the kelp bed at CBC; 2) urchin density at the front and fixed distances from the shifting position of the lower limit of the bed; 3) kelp biomass in the bed; and 4) temperature and wave height.

In June 2012, we established a linear series of benchmarks by setting into the bedrock 11 steel eyebolts at 1-m intervals in the barrens, ~3 m from the lower edge of the kelp bed. On 3 July, 2012, we attached one vinyl tape to the first benchmark of the series, and a second vinyl tape to the next benchmark. Both tapes were extended to the bed until they gave the same measure (±1 cm) when superimposed over the point marking the lower edge of the bed. This measure was subsequently converted by triangulation into a perpendicular distance between the lower edge of the bed and midway between the benchmarks. This procedure was repeated for each successive pair of benchmarks along the benchmark line, therefore yielding 10 absolute positions of the lower limit of kelp. Urchin density was measured in one quadrat (50 × 50 cm) placed at four distances along a transect line, which extended from the midpoint between each successive pair of benchmarks, up to ~3 m into the bed, for a total of 40 quadrats (four quadrats × 10 transects). The four distances were 1) 0.2 m from the benchmarks [hereafter termed “Barrens” zone]; 2) 2 m from the lower edge of the bed [“Pre-front” zone]; 3) at the leading edge of the urchin front [“Front” zone]; and 4) 2 m into the kelp bed [“Bed” zone]. Kelp biomass (wet weight of all sporophytes cut at the holdfast with a knife) was measured in five quadrats (50 × 50 cm) placed at 2-m intervals, ~2 m into the kelp bed. Accordingly, quadrats to measure urchin density in the Barrens zone were spatially fixed (0.2 m from the benchmarks). Quadrats to measure urchin density in the three other zones, as well as those to measure kelp biomass, shifted from one sampling event to the next because they were at fixed distances from the shifting edge of the kelp bed. These sampling procedures were repeated every 12 to 17 days until 25 September, 2012, for a total of seven sampling events although the last sampling event was excluded from the analysis (see Statistical analysis).

The water temperature at the study site was recorded every 30 min throughout the survey with a temperature logger with a precision of ±0.5°C (HOBO Pendant; Onset Computer Corporation) attached to one of the benchmark eyebolts. We followed the procedure established by Blain and Gagnon [36] to quantify the wave environment. The pressure of the water column on the seabed was recorded every two minutes by a water level logger with a precision of ±0.5 cm (HOBO U20-001-01-Ti Water Level Logger; Onset Computer Corporation). The logger was secured to the seabed, next to the line of benchmarks. Raw pressure values (psi) were corrected for barometric pressure by subtracting the hourly atmospheric pressure (psi) at the date and time of measurement (http://www.climate.weather.gc.ca/, Station St. John’s Intl A). Each corrected value was then converted into a raw water depth (m) by multiplying it by a conversion factor of 0.68 m psi^-1^ [40]. Raw water depths were corrected for tidal elevation and logger depth by subtracting the elevation at the date and time of measurement (http://www.tides.gc.ca/eng, Station 905) and the exact depth of the logger, yielding wave height. Temperature and significant wave height (SWH, the average height of the highest one-third of the wave data) were aggregated into mean daily averages, which we used to 1) study relationships between environmental variability and urchin density in the four zones; and 2) test the validity of thermal tipping ranges and regression equations from Experiment 1.

As mentioned, we calculated SWH from water pressure data acquired every two minutes. We used this relatively low frequency to avoid saturating the instrument’s data storage unit in between site visits (data content was downloaded every two to four weeks). To assess data quality, we measured, with the same instrument, water pressure every second for five hours on a day with moderate wave action. Raw pressure data were corrected and converted to SWH as per the procedure above. We then examined the correspondence among mean SWH calculated from data points taken: 1) every two minutes; 2) every minute; 3) every second during 10 min at 0.5-h intervals; and 4) every second during 10 min at 1-h intervals. The latter two sampling regimes, termed “burst sampling”, are commonly used in oceanographic studies [41, 42]. The positive and negative deviations of a particular wave were likely to cancel one another out in the high frequency (1 Hz) readings of the two burst sampling regimes. We eliminated this potential bias by using only the highest, positive heights of waves within each time interval. SWH was respectively 1) 0.227±0.049 [SD] m; 2) 0.232±0.052 m; 3) 0.233±0.054 m; and 4) 0.236±0.065 m. SWH from data acquired every two minutes was therefore ~4% lower than the largest estimate from data acquired every second during 10 min at 1-h intervals. Accordingly, we relied on data acquired every two minutes.

Pressure loggers similar to ours have been used to quantify wave regimes as an alternative to more accurate, yet costly devices such as acoustic current meters [28, 43, 44]. Yet, pressure signals from surface waves attenuate with depth in a frequency-dependent manner, with higher frequency wave signals attenuating more than lower frequency wave signals [45]. As a result, pressure sensors attached to the seabed (the present study) inevitably yield less accurate SWH estimates than pressure sensors at the sea surface. The three closest sources of SWH recorded at the sea surface during our study period are 100 to 470 km from CBC (S1 Table). These considerable distances, together with the offshore location of buoys and the obstruction to linear propagation of surface waves by land masses between buoys and CBC, could yield far less accurate estimates of SWH at CBC than those from our logger. Consequently, we chose not to construct the SWH climate at CBC with buoy data. We nevertheless used buoy data to provide a general indication of the ability of our pressure logger to detect changes in the magnitude of SWH. We found that SWH recorded at CBC correlated generally well with that recorded from the three buoys (S1 Table), and hence we used our SWH data to characterize the wave environment at CBC. Although this approach may underestimate SWH, it is arguably the most reliable we could use with the resources at hand.

### Statistical analysis

Note: details of the model parameters from the statistical analyses described below are provided in S2 Table.

Experiment 1 (individual feeding in water baths): Inspection of raw data (see Results) suggested feeding rate increased with temperature up to a breakpoint of ~12°C beyond which it decreased markedly, especially in large urchins. We used multiple piecewise (broken stick) regression to statistically detect the presence of a threshold temperature (n = 96). This type of regression is frequently employed to identify breakpoints in response variables with non-linear behaviors [46]. We applied the Gauss-Newton non-linear least-squares algorithm (with 100 iterations) with feeding rate as the response variable, and temperature (3, 6, 9, 12, 15, and 18°C) and urchin body size (t.d., mm) as independent, continuous variables. The model indeed converged at a temperature breakpoint of 12.0 (±1.1 SE)°C (see Results). Accordingly, we modeled feeding rate as a function of water temperature (°C) and urchin body size (t.d., mm) with multiple linear regression analyses [47], one with the observations at 3, 6, 9, and 12°C (n = 63), and one with the observations at 12, 15, and 18°C (n = 48). Both analyses were applied to the raw data.

Urchins used in the warmest temperature treatments, 15°C and 18°C, were exposed to potentially greater thermal shock than urchins in the colder temperature treatments. To test for potential biases in feeding rates due to thermal shock, we compared feeding rates of urchins exposed to the 15°C and 18°C treatments that had been maintained in the holding tanks at temperatures of no more than 6°C below their temperature treatment, to feeding rates of urchins exposed to the 15°C and 18°C treatments that had been maintained at temperatures >6°C below their temperature treatment. We used 6°C as the threshold temperature difference to form the two groups of comparison because 1) it is an accurate reflection of the average magnitude of sudden changes in sea temperature at BCC in early summer [see description of Experiment 1]; and 2) it captured the broadest range of feeding rates, from highest at 12°C to lowest at 18°C [see Results]. This procedure yielded 22 and 10 estimates of feeding rates for urchins that underwent a temperature difference of respectively >6°C and ≤6°C. We carried out a randomization (permutation) test [47] to test for a difference in feeding rates between both groups of urchins. We determined the probability of obtaining the observed difference between group means (D_*o*_ = -23.99 mg kelp urchin^-1^day^-1^) by calculating the proportion of values less than D_*o*_ (one-tailed test) in a frequency distribution of 1000 randomized differences. Randomized differences were generated by calculating the difference between means for two groups of data points (n = 22 and 10) drawn randomly from the 32 original estimates of feeding rates. We preferred this statistical approach over a Student’s t-test because it involves no assumption about the frequency distribution of the test statistic, and hence is a more robust approach to dealing with non-normal residuals and unequal sample sizes [47].

Experiment 2 (aggregative feeding in the wave tank): We used a two-way ANOVA with the factors Waves (null, low, intermediate, and high wave velocity) and Season (spring and summer) to examine temporal differences in the effect of wave action on the aggregative feeding rate of urchins on kelp (n = 8 [spring] and 7 [summer]). No transformation corrected the heteroscedasticity of the residuals in the analysis on the raw data. Therefore, the ANOVA was also run with the rank-transformed data. Because both analyses gave similar conclusions about the significance of each factor, we presented the results from analyses on the raw data [48]. Prior to running this two-way ANOVA, we had used two one-way ANOVAs, one for each season, with the factor Block (each of the eight spring or seven summer blocks of four days during which one replicate of each treatment was done), to determine whether results differed among blocks of days in each season. There was no significant effect of the factor Block in spring (F_7,24_ = 0.47; *p* = 0.85) and summer (F_6,21_ = 1.91; *p* = 0.11), and hence we ran the two-way ANOVA on data pooled from all blocks. We used a two-way MANOVA [49] with the factors Waves (null, low, intermediate, and high wave velocity) and Season (spring and summer) to examine temporal differences in the effect of wave action on the proportion of urchins (out of 118) feeding on kelp, underneath the kelp canopy, on the tiles outside of the area swept by kelp, and on the tank walls at the end of trials (n = 8 [spring] and 7 [summer]). The data were logit-transformed [50] to correct for heteroscedasticity of the residuals in the analysis on the raw data.

Field observations: We used linear regression analysis to examine relationships between urchin density and mean sea temperature (Temp) and significant wave height (SWH) at CBC. We used temperature and SWH data averaged over the 48 hours preceding each sampling event because preliminary analysis showed stabilization of variation beyond 48 h. We began with a multiple regression model with the factors Temp, SWH, and Zone (a categorical variable representing the four sampling zones: Barrens, Pre-front, Front, and Bed) to determine if sea temperature and SWH had an effect on urchin density across the zones (n = 24). Temperature was the only factor affecting density across the zones (see Results). We therefore used simple linear regression analysis with the factor Temp to determine the relationship between sea temperature and urchin density in each of the four zones separately. We excluded data acquired on 25 September, 2012 (14 days after the passage of the tail end of Hurricane Leslie) from all regression analyses because they differed markedly from the rest of the dataset (see Results). Sea temperature and SWH from 3 July to 25 September, 2012, were not correlated (Pearson’s product-moment correlation, *r* = -0.169, *p* = 0.087), which enabled testing effects of both environmental factors. Each data point in each regression was based on mean urchin density calculated from all quadrats in each zone on each of the six sampling events from 3 July to 13 September, 2012 (n = 24 and 6 for multiple and simple regression analyses, respectively). All regressions were applied to the raw data. As mentioned previously, quadrats to measure urchin density in the Barrens zone were spatially fixed, whereas those in the three other zones shifted from one sampling event to the next. Analysis of residuals from the multiple linear regression analysis and four simple linear regression analyses confirmed that data were not autocorrelated (S3 Table).

We tested the hypothesis that water temperature can predict short-term kelp bed destruction by *S*. *droebachiensis* in calm hydrodynamic environments by comparing expected and observed rates of kelp loss (g kelp day^-1^) at CBC. Expected rates were calculated with the equations derived from Experiment 1, whereas observed rates came from our observational dataset at CBC. The following procedure was used to determine the expected daily rate of kelp loss for each of the six time intervals available from 3 July to 25 September, 2012. We calculated mean sea temperature for the time interval to determine which of the two regression equations (see Results) to use to calculate the daily feeding rate per urchin. We applied the appropriate equation a first time by assigning mean sea temperature and 25 mm to the temperature (T) and urchin size (S) terms, respectively. We ran it again with mean sea temperature and 45 mm, therefore providing one rate for small and one rate for large, urchins. For consistency, we used the lowest urchin size in each size category permitted by the limits of inference of the equation. Logistical considerations precluded measuring the size of urchins at CBC. The only data of urchin abundance and size structure in fronts for the region of Newfoundland that we are aware of had insufficient resolution to serve our goal [39, 51]. We therefore determined the likely numbers of small and large urchins that together made up the total number of urchins at the front. This was done by multiplying mean urchin density at the front by the proportion of small (0.65) and large (0.35) urchins in fronts at the lower limit of *A*. *esculenta* beds at a similar time of the year in the Mingan Islands in the northern Gulf of St. Lawrence [24]. We used published data from the Mingan Islands because this is the nearest system with comparable urchin densities and drivers of urchin-kelp bed dynamics [24]. Resulting numbers of small and large urchins were then multiplied by corresponding feeding rates obtained from the regression equations (Experiment 1) and summed to obtain the expected total daily rate of kelp loss for the interval. The following procedure was used to determine the observed daily rate of kelp loss in each of the six intervals. We multiplied the mean surface area over which the lower edge of the kelp bed shifted during the interval along a 1-m swath of seabed by the kelp biomass averaged from measurements on the two sampling days that formed the interval. The resulting value was then divided by the number of days in the interval. We used simple linear regression analysis to measure the fit between expected and observed daily rates of kelp loss. The analysis was applied to the raw data (n = 6). We did not attempt to correct the expected daily rates of kelp loss with the equations from Experiment 2 because of data incompatibility from the different approaches used to quantify the wave environment: horizontal wave velocity (in m s^-1^) in the lab and amplitude of the vertical displacement of the sea surface (SWH, in meters) at CBC.

In all ANOVAs, MANOVA, and regression analyses, homogeneity of the variance was verified by examining the distribution of the residuals. Normality of the residuals was verified by examining the normal probability plot of the residuals [52]. To detect differences among levels within a factor (ANOVAs and MANOVA), we used Tukey HSD multiple comparison tests (comparisons based on least-square means) [47]. When a factor, or interaction between factors, was significant in the MANOVA, we examined the univariate model for the response variable to identify which one(s) contributed to the multivariate effect. This was done by conducting an ANOVA for the response variable with the same factors as in the MANOVA. The Pillai's trace multivariate statistic, which is more robust than other multivariate statistics to deviations from homoscedasticity and normality of the residuals, as well as more conservative with small and uneven sample sizes, was used in the MANOVA to determine which factor(s) with more than two levels was statistically significant [49]. Because we could not presume of the absence or presence of synergistic effects between explanatory variables, all multiple linear regression analyses were conducted using the multiplicative error model approach, which tests for individual and interactive effects of the explanatory variables [53]. Accordingly, when interactive effects were not significant, we presented models with individual effects of only those explanatory variables that were significant in the truncated model. A significance level of 0.05 was used. All analyses were conducted with JMP 7.0 and Minitab 16.2.4.

## RESULTS

### Experiment 1

**Fig 2:**
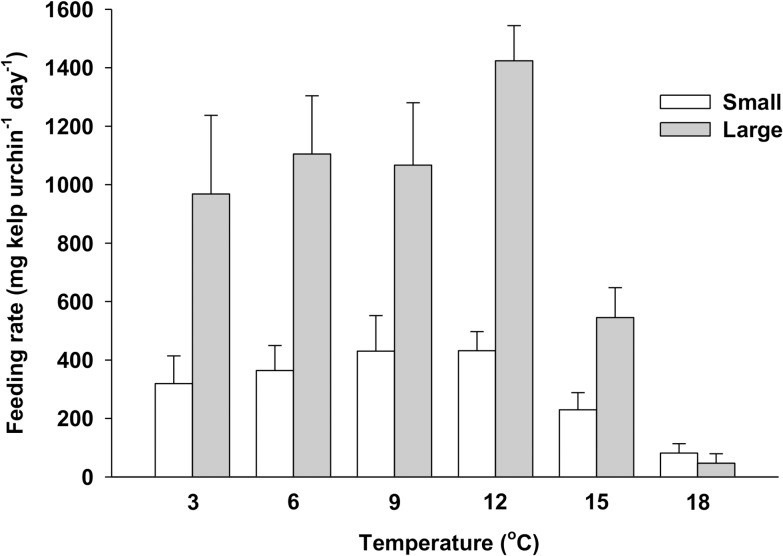
Mean (+SE) feeding rate of small (25–35 mm t.d.) and large (45–60 mm t.d.) green sea urchins (*Strongylocentrotus droebachiensis*) on kelp (*Alaria esculenta*) in seawater at 3, 6, 9, 12, 15, and 18°C (Experiment 1).

Inspection of data from Experiment 1 suggested that individual urchin feeding on kelp varied with body size among the six temperature treatments (Fig. 2). Feeding generally increased across the 3–12°C range in both small and large urchins. However, it was 2.5 (9°C) to 3.3 (12°C) times higher in large than small urchins for a given temperature, and peaked to 1424 (±120, SE) mg kelp urchin^-1^ day^-1^ in large urchins at 12°C (Fig. 2). Increasing temperature above 12°C negatively affected large urchins as shown by the 62% and 91% drops in feeding from 12°C to 15°C, and from 15°C to 18°C, respectively, i.e. a difference of two orders of magnitude between 12°C and 18°C (Fig. 2). Feeding in small urchins at 15 and 18°C was comparable to that in large urchins, while remaining as low (<432 [±59] mg kelp urchin^-1^ day^-1^) as that in small urchins at any of the other temperatures (Fig. 2). Piecewise and multiple linear regressions revealed a temperature breakpoint of 12.0 (±1.1)°C, below and above which urchin feeding was respectively positively and negatively correlated with temperature and body size (Table 1). The mean feeding rate of urchins exposed to the warmest temperature treatments, 15°C and 18°C, and previously maintained in the holding tanks at temperatures ≤6°C below their temperature treatment (242.3±51.1 mg kelp urchin^-1^ day^-1^), was similar to that of urchins exposed to the 15°C and 18°C treatments and previously maintained at temperatures >6°C below their temperature treatment, 218.3±101.9 mg kelp urchin^-1^ day^-1^ (randomization test; *p* = 0.365). Thermal shock, if present, was therefore unlikely to cause the drop in feeding rates above 12°C.

**Table 1:** Results of multiple linear regression analyses examining relationships between feeding rate of green sea urchins (*Strongylocentrotus droebachiensis*) on kelp (*Alaria esculenta*), and water temperature (T) and urchin body size (S) (test diameter [t.d.], which can take on values from 25 to 60 mm) in each of two temperature ranges in Experiment 1 (see Materials and methods for determination of the temperature breakpoint [12°C] delimiting the temperature ranges).

Temperature range	Equation for feeding rate (mg kelp urchin^-1^ day^-1^)	r^2^	F _(df)_	*p*
[3–12]°C	-814.9 +36.8*T + 31.6*S	0.513	31.66 _(2,60)_	<0.001
[12–18]°C	-2363.9 + 140.9*T + 122.0*S—7.0*T*S	0.840	77.25 _(3,44)_	<0.001

### Experiment 2

**Fig 3:**
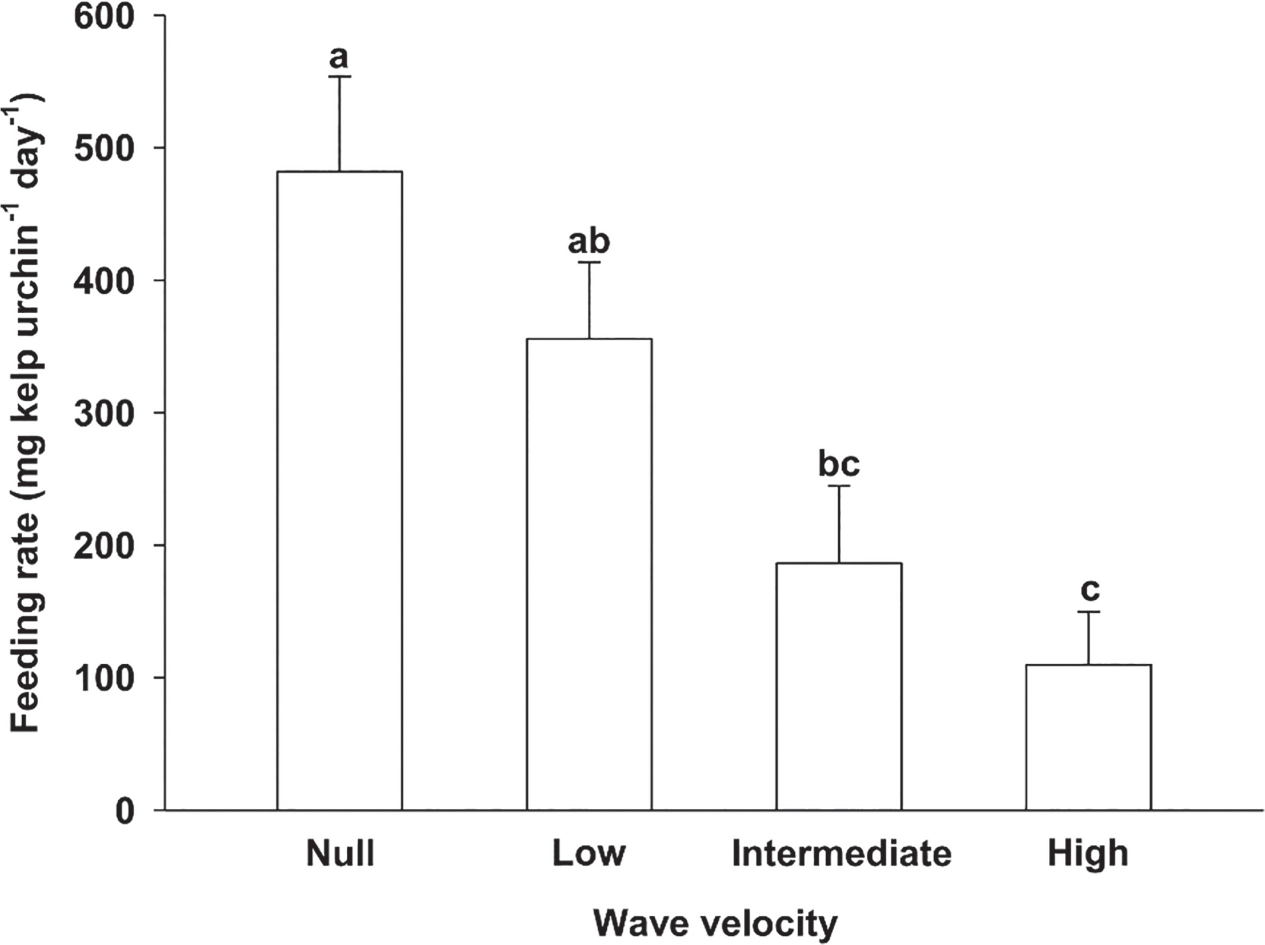
Mean (+SE) feeding rate of large (40–60 mm t.d.) green sea urchins (*Strongylocentrotus droebachiensis*) on kelp (*Alaria esculenta*) at null, low, intermediate, and high wave velocity (0.0, 0.1, 0.2, and 0.3 m s^-1^, respectively) (Experiment 2). Data were pooled across Season (spring and summer) treatments. Bars not sharing the same letter are different (LS means tests, *p<*0.05; n = 15 for each velocity).

Analysis of data from Experiment 2 indicated that aggregative urchin feeding on kelp varied among the four wave velocities independently of season (Table 2). Feeding rate peaked to 482 (±72) mg kelp urchin^-1^ day^-1^ in the absence of waves, being at least 2.5 times higher than at intermediate (0.2 m s^-1^) and high (0.3 m s^-1^) velocities (Fig. 3). Increasing velocity from null to low (0.1 m s^-1^) had no perceptible effect on feeding as shown by a non-significant drop of 26% (Fig. 3). The overall (pooled across wave velocities) feeding rate during summer (362 [±59] mg kelp urchin^-1^ day^-1^), when water temperature averaged 13.9±0.4°C, was 1.7 times higher than during spring (215 [±34] mg kelp urchin^-1^ day^-1^), when temperature was 5.1±0.2°C (a significant difference, Table 2).

**Table 2:** Summary of two-way ANOVA (applied to raw data) examining the effect of Waves (null, low, intermediate, and high wave velocity) and Season (spring and summer) on feeding rate of green sea urchins (*Strongylocentrotus droebachiensis*) on kelp (*Alaria esculenta*) in Experiment 2 (see Materials and methods for a description of the experiment).

Source of variation	*df*	MS	*F*-value	*p*
Waves	3	4.24 x 10^5^	8.90	<0.001
Season	1	3.23 x 10^5^	6.79	0.012
Waves × Season	3	1.25 x 10^4^	0.26	0.852
Error	52	4.50 x 10^4^		
Corrected total	59			

**Fig 4:**
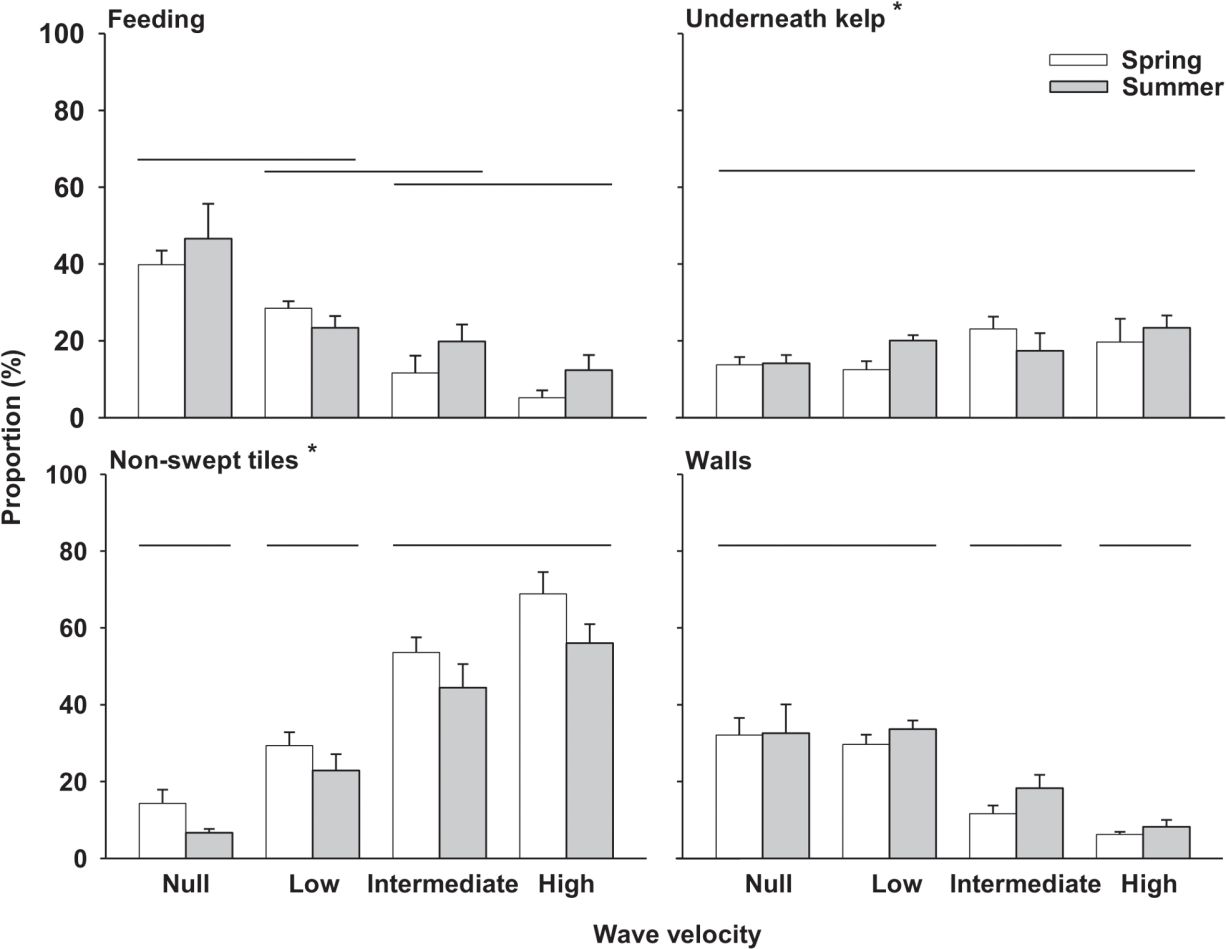
Proportion (+SE) of large (40–60 mm t.d.) green sea urchins (*Strongylocentrotus droebachiensis*) feeding on kelp (*Alaria esculenta*), underneath the kelp canopy, on the tiles outside of the area swept by kelp, and on the tank walls, at the end of trials at null, low, intermediate, and high wave velocity (0.0, 0.1, 0.2, and 0.3 m s^-1^, respectively) in two seasons (Spring and Summer) (Experiment 2). Wave treatments not bracketed by the same horizontal line are different (data pooled across seasons, LS means, *p*<0.05, n = 15 for each velocity). Panels with an asterisk indicate a significant difference in proportions between seasons (Summer>Spring for urchins underneath the kelp canopy and Spring>Summer for urchins on non-swept tiles) (data pooled across wave velocities, LS means, *p*<0.05, n = 32 and n = 28 for Spring and Summer, respectively).

The MANOVA examination showed that wave velocity and season independently affected the proportion of urchins feeding on kelp and displacing in the wave tank (Table 3). The proportion of urchins feeding decreased steadily with an increase in wave velocity, from null (43%) to high (8%) (i.e. a fivefold decrease; LS means, *p<*0.001), while being similar in spring (21%) and summer (26%) (LS means, *p* = 0.136) (Table 4, Fig. 4). Conversely, wave velocity had no perceptible effect on the number of urchins that had moved underneath the kelp canopy and urchins that did so were significantly more numerous in summer (19%) than spring (17%) (Table 4, Fig. 4). Wave velocity and season independently affected the proportion of urchins that remained on the tiles outside of the area swept by kelp, with as little as 11% in the absence of waves to up to 63% at high velocity, and 42% and 33% in spring and summer, respectively (Table 4, Fig. 4). The proportion of urchins that climbed on the tank walls varied with wave velocity only, being similar (~32%) at null and low velocities (LS means, *p* = 0.701), and decreasing to 7% with increasing velocity above 0.1 m s^-1^ (Table 4, Fig. 4).

**Table 3:** Summary of two-way MANOVA (applied to logit-transformed data) examining the effect of Waves (null, low, intermediate, and high wave velocity) and Season (spring and summer) on the proportion of green sea urchins (*Strongylocentrotus droebachiensis*) feeding on kelp (*Alaria esculenta*), underneath the kelp canopy, on the tiles outside of the area swept by kelp, and on the tank walls, at the end of trials in Experiment 2 (see Materials and methods for a description of the experiment).

Source of variation	Test	Value	*F*-value	NumDF	DenDF	*p*
Waves	Pillai’s Trace	1.27	9.36	12	153	<0.001
Season	F Test	0.27	3.30	4	49	0.018
Waves × Season	Pillai’s Trace	0.28	1.31	12	153	0.217

**Table 4:** Summary of two-way ANOVAs (applied to logit-transformed data) examining the effect of Waves (null, low, intermediate, and high wave velocity) and Season (spring and summer) on the proportion of green sea urchins (*Strongylocentrotus droebachiensis*) feeding on kelp (*Alaria esculenta*), underneath the kelp canopy, on the tiles outside of the area swept by kelp, and on the tank walls, at the end of trials in Experiment 2 (see Materials and methods for a description of the experiment).

Activity or location	Source of variation	*df*	MS	*F*-value	*p*
Feeding	Waves	3	3.12	22.18	<0.001
	Season	1	0.35	2.42	0.126
	Waves × Season	3	0.18	1.28	0.290
	Error	52	0.14		
	Corrected total	59			
Underneath the kelp	Waves	3	0.08	1.55	0.214
canopy	Season	1	0.21	4.09	0.048
	Waves × Season	3	0.10	1.97	0.130
	Error	52	0.05		
	Corrected total	59			
On the tiles outside of the	Waves	3	4.83	51.51	<0.001
area swept by kelp	Season	1	0.73	7.81	0.007
	Waves × Season	3	0.01	0.12	0.949
	Error	52	0.09		
	Corrected total	59			
On the tank walls	Waves	3	2.29	26.32	<0.001
	Season	1	0.08	0.92	0.343
	Waves × Season	3	0.06	0.68	0.566
	Error	52	0.09		
	Corrected total	59			

### Field observations

**Fig 5:**
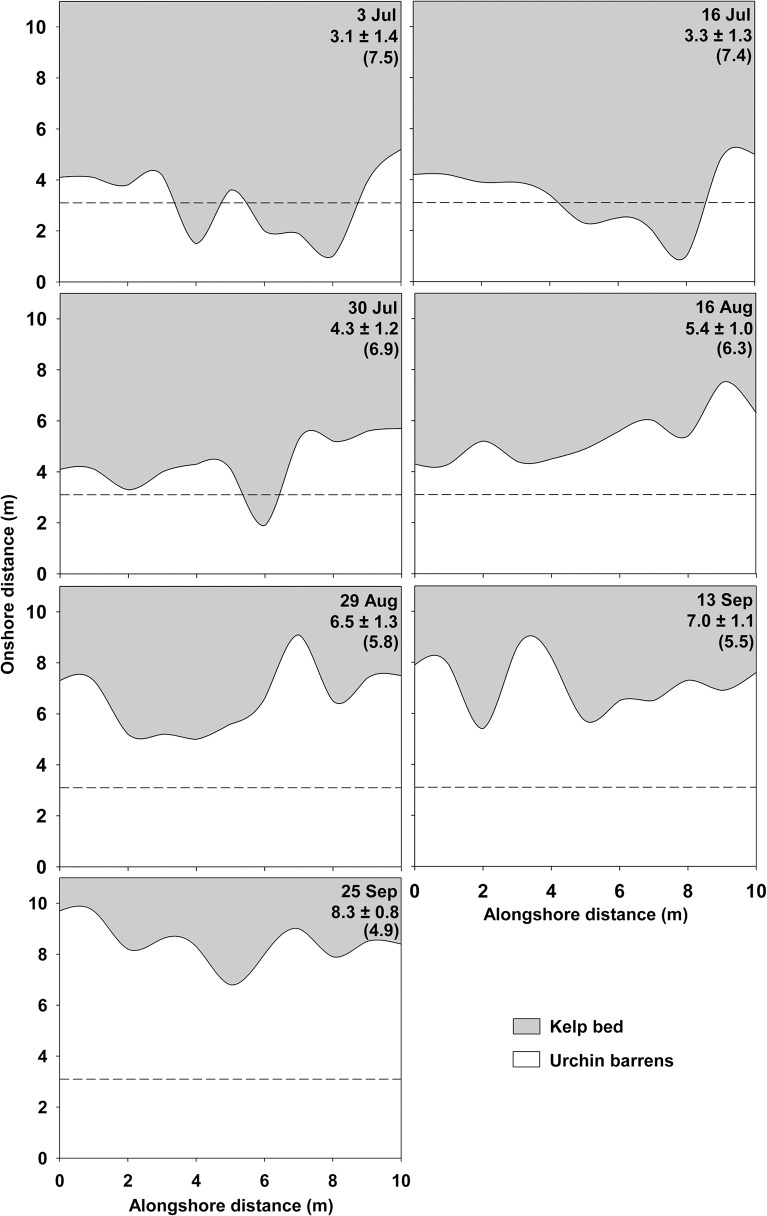
Change in the position of the kelp-barrens interface at Cape Boone Cove from 3 July to 25 September, 2012. Values directly below sampling dates are the mean distance (±SE) of the kelp-barrens interface relative to benchmark eyebolts in the urchin barrens (0 m). The depth across the grid (from 10 to 0 m along the y-axis) is from 4 to 9 m. Values in parentheses are the approximate depth (in m) of the kelp-barrens interface. Horizontal dashed lines indicate the mean distance of the kelp-barrens interface on the first sampling event (3 July).

**Fig 6:**
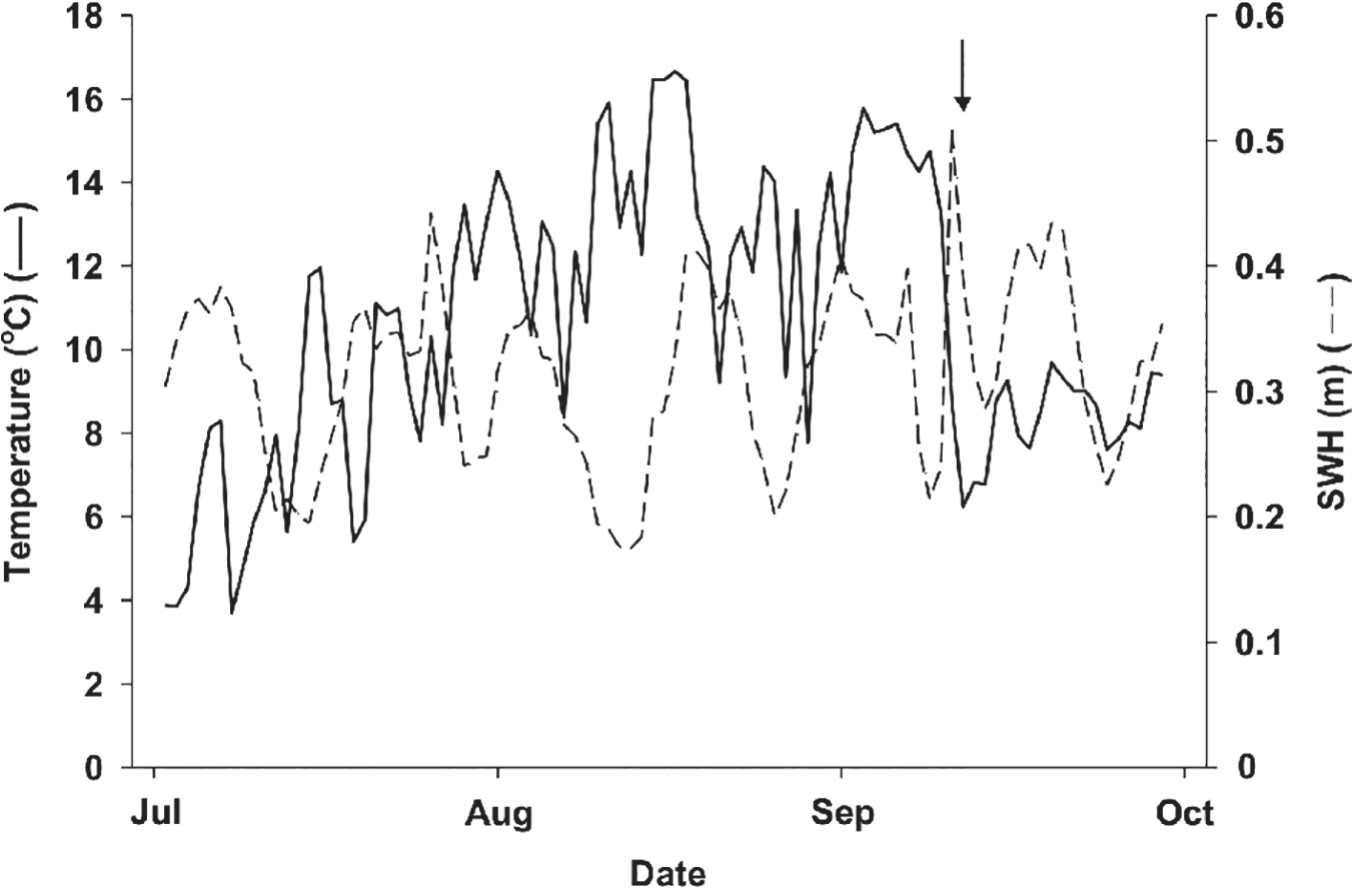
Change in mean daily sea temperature and significant wave height (SWH) at Cape Boone Cove from 1 July to 30 September, 2012. Sea temperature and wave height data were acquired every 30 and 2 minutes, respectively, with one temperature logger and one water level logger secured to the seabed at a depth of 9 m. The arrow indicates the date (11 September) that the tail end of Hurricane Leslie reached the southeastern tip of Newfoundland (note the sharp decline in sea temperature and slight increase in SWH associated with this event).

The lower limit of the kelp bed at CBC retreated on average by 0.43 m week^-1^ (1.84 m month^-1^) from 3 July to 25 September, 2012, and by the end had been pushed back by ~5.2 m (Fig. 5). The urchin front moved from a depth of ~7.5 m at the beginning of July to a depth of ~4.9 m in late September (Fig. 5). Kelp biomass within the first 2 m above the lower edge of the bed was relatively constant at 3.1 ± 0.3 kg m^-2^ from 3 July to 13 September. It was largely dominated (>90%) by *A*. *esculenta*. On 25 September (the last sampling day), we noted a shift in dominance from *A*. *esculenta* to larger and heavier *Laminaria digitata* sporophytes (~60%). Kelp biomass then peaked at 5.4±1.3 kg m^-2^ and urchins had grazed through much of the lower portion of the bed dominated by *A*. *esculenta*. The passage of the tail end of Hurricane Leslie on 11 September coincided with a sudden drop in sea temperature from ~15°C to ~6°C, as well as a twofold increase in SWH that did not exceed 0.51 m (Fig. 6). Temperature remained relatively low, below ~9°C, until the end of the survey, whereas SWH returned to the general pattern of variation between ~0.2 and ~0.4 m seen before Leslie (Fig. 6). That SWH did not exceed ~0.5 m even during the passage of the tail end of Leslie speaks to the relatively mild wave environment at CBC throughout the survey (Leslie had considerably weakened by the time it reached our site). Nevertheless, the sudden changes in sea temperature and state that accompanied Leslie seemed to adversely affect *A*. *esculenta*, which was already showing signs of tissue damage prior to the storm. We saw large pieces of *A*. *esculenta* blades covering the higher end of the barrens two days after the hurricane, as well as broken stipes of *A*. *esculenta* without blades two and 14 days after the hurricane. In contrast, *L*. *digitata* sporophytes remained generally healthy throughout the survey.

**Fig 7:**
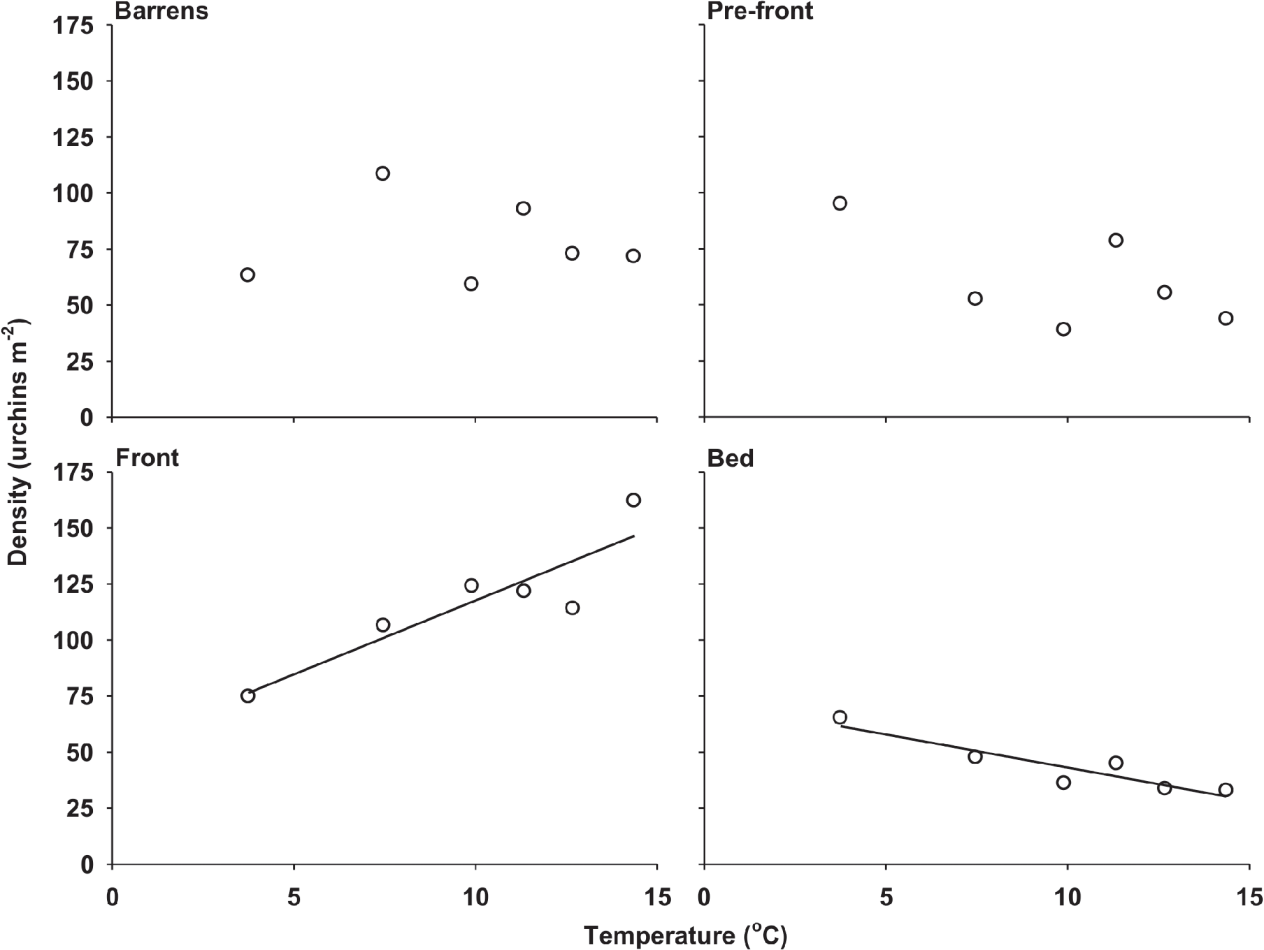
Relationship between the density of green sea urchins (*Strongylocentrotus droebachiensis*) and sea temperature in each of the four zones sampled at Cape Boone Cove from 3 July to 13 September, 2012. Barrens: 0.2 m from benchmark eyebolts in the urchin barrens; Pre-front: 2 m from the lower edge of the kelp bed; Front: at the leading edge of the urchin front; and Bed: 2 m into the kelp bed (see Table 6 for details of the regression analyses).

Urchin density was consistently higher at the front (Front zone) than in any of the three other zones (Barrens, Pre-front, and Bed). It peaked at 162.0 ± 22.7 individuals m^-2^ on 16 August, when mean sea temperature also peaked at 16.7°C (Fig. 6). Multiple linear regression analysis showed that urchin density across zones from 3 July to 13 September, 2012, was affected by sea temperature but not significant wave height (SWH) (Table 5). Simple linear regression analysis indicated that density in the Front and Bed was positively and negatively related to sea temperature, respectively (Table 6, Fig. 7). The magnitude of the effect of sea temperature on urchin density was greatest in the Front: density increased by a factor of 6.6 for every degree increase in temperature (Table 6). This effect was twice more pronounced than that observed in the bed, where density decreased in a 3:1 ratio with temperature (Table 6). Temperature had no detectable effect on urchin density in the Barrens and Pre-front (Table 6, Fig. 7).

**Table 5:** Summary of multiple linear regression analysis (applied to raw data) examining the effect of sea temperature (Temp) and significant wave height (SWH) on the density of green sea urchins (*Strongylocentrotus droebachiensis*) in the four zones (Zone, a categorical variable: Barrens, Pre-front, Front, and Bed) sampled at Cape Boone Cove from 3 July to 13 September, 2012.

Source of variation	*df*	MS	*F*-value	*p*
Temp	1	39.50	0.17	0.689
SWH	1	154.92	0.66	0.433
Zone	3	358.39	1.52	0.260
SWH*Zone	3	394.25	1.67	0.225
Temp*Zone	3	1368.46	5.81	0.011
Error	12	235.68		
Corrected total	23			

**Table 6:** Summary of simple linear regression analyses (applied to raw data) examining the relationship between the density of green sea urchins (*Strongylocentrotus droebachiensis*) and sea temperature (Temp [in°C], the slope parameter) in each of the four zones sampled at Cape Boone Cove from 3 July to 13 September, 2012.

Zone	Intercept (95% CL)	Temp (95% CL)	r^2^	*F*_(df)_	*p*
Barrens	78.6 (6.6, 150.6)	-0.1 (-6.9, 6.8)	0.0002	0.001 _(1,4)_	0.981
Pre-front	95.3 (30.1, 160.4)	-3.5 (-9.7, 2.7)	0.381	2.46 _(1,4)_	0.192
Front	51.7 (3.4, 100.0)	6.6 (2.0, 11.2)	0.799	15.93 _(1,4)_	0.016
Bed	72.6 (54.1, 91.1)	-3.0 (-4.7, -1.2)	0.844	21.70 _(1,4)_	0.010

Barrens: 0.2 m from benchmark eyebolts in the urchin barrens; Pre-front: 2 m from the lower edge of the kelp bed; Front: at the leading edge of the urchin front; and Bed: 2 m into the kelp bed. Model coefficients are shown with corresponding 95% confidence limits (CL).

**Fig 8:**
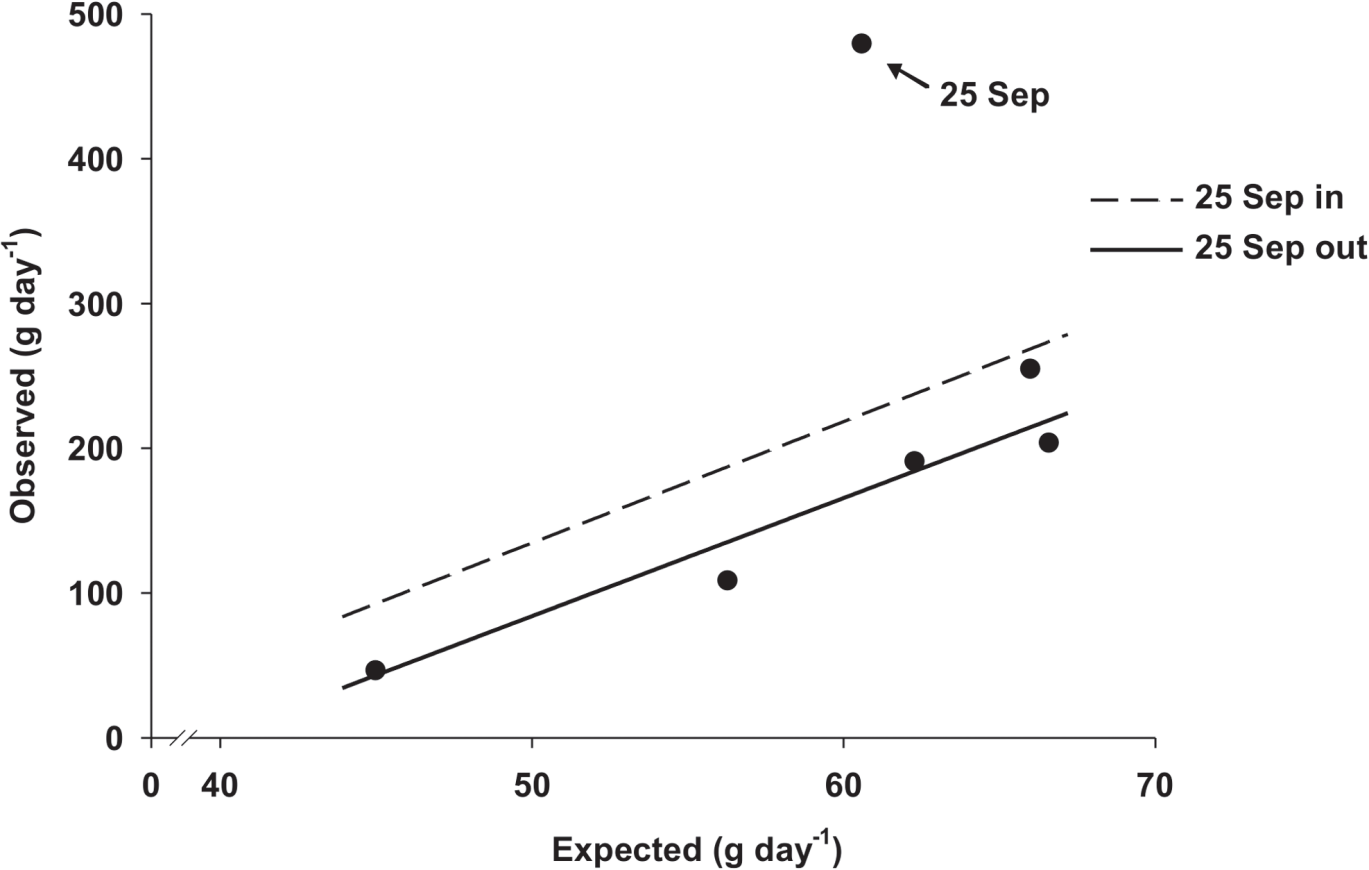
Relationship between observed and expected daily rates of kelp loss during the summer 2012 survey at Cape Boone Cove (CBC) with and without data from 25 September, the last sampling event, which was 14 days after the passage of the tail end of Hurricane Leslie. Observed rates were calculated from our observational dataset at CBC, whereas expected rates were calculated with the equations derived from Experiment 1 (see Statistical analysis and Table 1 for details of the observational dataset and equations used and Table 7 for details of the two regression lines shown).

**Fig 9:**
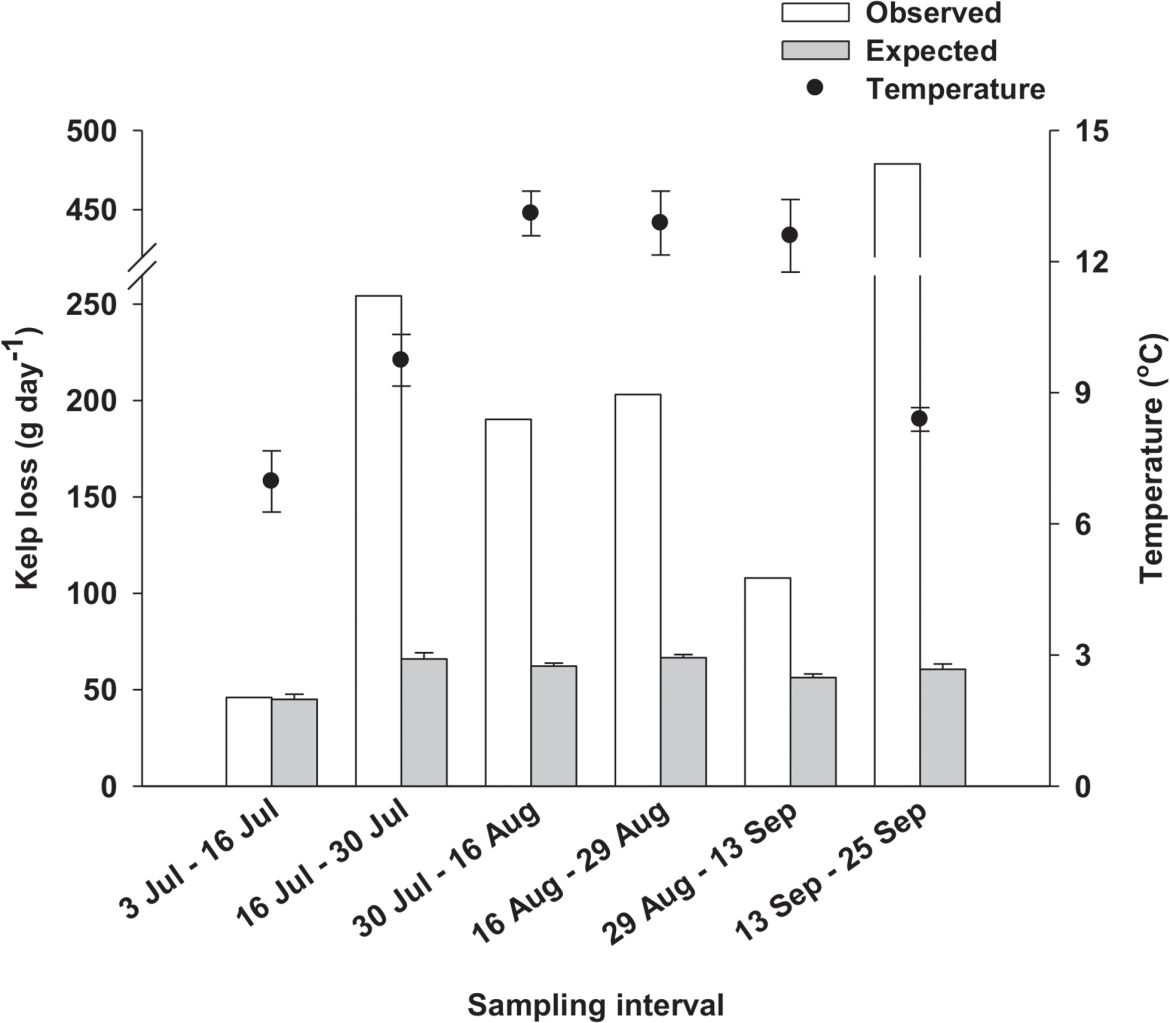
Observed and expected (+SE) daily rates of kelp loss and mean (±SE) sea temperature for each of the six sampling intervals during the summer 2012 survey at Cape Boone Cove. Sampling intervals 5 (29 Aug—13 Sep) and 6 (13 Sep—25 Sep) include data acquired two and 14 days (on 13 and 25 September) after the passage of the tail end of Hurricane Leslie.

There was a strong (r^2^ = 0.878) positive relationship between observed and expected daily rates of kelp loss at CBC from 3 July to 13 September, 2012 (Table 7, Fig. 8). Yet, the slope of this relationship, 8.8 (Table 7), was similar to that of a theoretical relationship in which observed rates increase 10 times faster than expected rates (paired t-test, t_3_ = -0.75, *p* = 0.510; Fig. 8), therefore indicating that the observed rates of kelp loss were one order of magnitude greater than those expected. Including data from 25 September, the last sampling event (14 days after Hurricane Leslie), only marginally affected the slope (9.9) of the relationship, which, however, was not significant (Table 7). Observed rates of kelp loss increased with mean sea temperature in July, but leveled off in August and decreased during the first part of September when temperature reached and remained within the 12–15°C tipping range found in Experiment 1 (Fig. 9).

**Table 7:** Summary of simple linear regression analyses (applied to raw data) examining relationships between observed and expected daily rates of kelp loss during the summer 2012 survey at Cape Boone Cove with and without data from 25 September (the last sampling event).

Data	Intercept (95% CL)	Slope (95% CL)	r^2^	*F*_(df)_	*p*
25 Sep in	-374.1 (-1688.0, 940.0)	9.9 (-12.1, 31.8)	0.281	1.56 _(1,4)_	0.279
25 Sep out	-361.2 (-667.8, -54.7)	8.8 (3.7, 13.9)	0.878	29.85 _(1,3)_	0.012

Model coefficients are shown with corresponding 95% confidence limits (CL).

## DISCUSSION

Our study demonstrates that sea temperature, and not only hydrodynamic forces as found in other studies [28–30], can predict short-term kelp bed destruction by urchin fronts in shallow reef communities. We experimentally determined that individual feeding in the green sea urchin, *Strongylocentrotus droebachiensis*, during early summer (June-July) obeyed a non-linear, size- and temperature-dependent relationship. Feeding in large (45–60 mm t.d.) urchins was consistently highest at temperatures <12°C and dropped sharply within and above the 12–15°C range (Experiment 1). We also found that daily rates of kelp loss over ~3 months at the Cape Boone Cove (CBC) site were highly correlated (88%) with those calculated from sea temperature at the site and regression equations derived from results of the latter experiment. These findings speak to the importance of considering body size and natural variation in sea temperature in studies of urchin-kelp interactions. They also provide a mechanistic explanation for temporal variation in urchin-kelp interfaces in environments dominated by low hydrodynamic forces.

Most studies of kelp-bed boundary dynamics in the northwestern Atlantic (NWA) establish statistical relationships among rates of kelp retreat, urchin density or biomass at fronts, sea temperature, and significant wave height (SWH) [22–25, 28–30]. These studies have yielded inconsistent outcomes from strong correlations to contradictory results. Use of urchin density or biomass as proxies of the destructive potential of urchin fronts may partly account for discrepancies among studies. Certainly, the amount of kelp a front eradicates is fundamentally determined by the ability of each individual in the front to consume kelp. Seminal studies of ecological energetics in green sea urchins [54–57], together with more recent studies of gonadic growth and feed intake in aquaculture settings [58, 59], support the notion that feeding increases with body size and temperature under conditions of low water motion. Predicting kelp bed destruction from urchin density or biomass without considering the size structure of urchins at the front and sea temperature may therefore overlook per capita aspects of urchin-kelp interactions that ultimately drive a front’s performance.

Metabolic rate in most animals and plants, including sea urchins, is governed largely by two interacting processes: 1) the temperature dependence of biochemical processes by which metabolic rate accelerates with increasing temperature within a biologically relevant temperature range; and 2) the quarter-power allometric relation by which biological rate processes scale with body size [32]. As per the emerging conceptual foundations of the metabolic theory of ecology (MTE), these organismal processes dictate physiological performance, rates of resource acquisition, growth, reproduction, and survival, which in turn affect processes at the species, population, community, and ecosystem levels [19, 31, 60, 61]. Recent studies suggest that some marine herbivore-plant interactions strengthen with warming. For example, Poore et al. [62] found that increasing temperature reduced survival and growth of the amphipod *Peramphithoe parmerong*, while affecting the palatability of its algal food, *Sargassum linearifolium*. O’Connor [63] showed that increasing temperature increased per capita interaction strength between the amphipod *Ampithoe longimana* and seaweed *Sargassum fillipendula*, while reversing a positive effect of temperature on growth in the latter.

Our results generally support the tenets of the MTE by showing that individual and aggregative feeding in *S*. *droebachiensis*, and ultimately urchin-kelp interactions in a natural habitat, are influenced by water temperature and urchin body size (see details below). They also provide new insights into the biologically relevant temperature range for *S*. *droebachiensis* during summer at our study site, when sea temperature typically drops and rises by up to 10°C over the course of only a few hours to days [35, 36]. Experiment 1 established that kelp consumption by urchins in the laboratory increased linearly with temperature across the 3–12°C range and dropped markedly within and above the 12–15°C range. This relationship was more apparent in large than small urchins, which has three important conceptual implications. Firstly, it suggests that below ~12°C, the rate of kelp loss to a front should be partly driven by differences in the size structure of urchins, with an overriding influence, per capita, of large urchins. This means, for example, that small (≤4 cm t.d.) urchins, which represent on average ~65% of urchins at fronts advancing over *A*. *esculenta* beds during summer in the northern Gulf of St. Lawrence [24], may cause ~43% of the kelp loss, compared to ~57% of the loss by ~53% fewer, albeit larger (>4 cm t.d.), urchins. Secondly, it shows that feeding is more sensitive to increases in temperature above ~12°C in large than small urchins. Accordingly, the rate of kelp loss to a front within and above the 12–15°C range should be negatively related to sea temperature at sites with low hydrodynamic forces. It should also be increasingly predictable upon strict knowledge of the number of urchins (density) at the front, i.e. regardless of size structure. Thirdly, it establishes that feeding in *S*. *droebachiensis* is much reduced at temperatures above 15°C, a drop that we showed was unlikely to be caused by thermal shock as urchins were introduced to their experimental temperatures. Kelp loss to a front should therefore slow down as temperature approaches 18°C, although such relatively high temperatures (and other factors) may also increase kelp mortality, yielding similar or higher overall rates of kelp loss (see below). The 12–15°C feeding discontinuity documented herein is based on urchins that were not pre-acclimated to their respective experimental temperature treatments. This procedure was used to account for effects of natural variability in sea temperature at CBC on urchin feeding. It is therefore a good representation of the ability of urchins to adapt feeding to relatively sharp temperature changes (in the present study up to ~10°C) during a specific period (June-September). Longer-term studies are required to determine if the 12–15°C feeding discontinuity changes in space and time. Such a discontinuity was nevertheless useful in predicting patterns of kelp loss at CBC (see below). Certain marine invertebrates such as limpets and mussels physiologically adapt, to various degrees, to acute and long-term rises in temperature by adjusting the expression of protein-coding genes [64–66]. Addressing the extent to which the green sea urchin may physiologically adapt to acute and long-term changes in sea temperature is critical to define the thermal boundaries within which destructive grazing of kelp communities is likely to occur as the global ocean continues to warm [16, 17]. It would also help further test the limits of prediction of the MTE by incorporating effects of short-term variability (in addition to long-term mean changes) in sea temperature on urchin feeding and the ecological cascades which may result from it.

Results of Experiment 1 were consistent with patterns in the field as shown by the high statistical fit between observed and expected daily rates of kelp loss at CBC from 3 July to 13 September, 2012, when sea temperature varied between 3.7 and 16.7°C and SWH was consistently low, <0.51 m. Observed rates of kelp loss increased with mean sea temperature in July, but leveled off in August and decreased during the first part of September when temperature reached and remained within the 12–15°C tipping range found in Experiment 1. Interestingly, the observed rates of kelp loss were approximately one order of magnitude greater than those expected. The latter result is a priori surprising given that the expected rates were obtained from results of Experiment 1, in which urchin feeding was not impeded by factors such as water turbulence and competition. Expected rates should therefore have been equal or higher than the observed rates. The lower portion of the kelp bed at CBC was composed mainly of *Alaria esculenta*, which started to erode in the first two weeks of August, when sea temperature on many days exceeded 12°C and hovered around the proposed lethal 16°C for the species [67]. We think that the observed rates of kelp loss from mid-August to mid-September (prior to Hurricane Leslie) originated from a temperature-induced decline in urchin feeding offset by increasing kelp loss through natural senescence. The sudden 4-fold increase in the rate of kelp loss over the two weeks that followed Leslie was largely driven by fragmentation and detachment of the weakened *A*. *esculenta* sporophytes. The urchin front progressively moved into shallower water as it grazed down the lower margin of the kelp bed, and hence it may have been exposed to increasingly greater hydrodynamic forces resulting from lesser attenuation of wave motion at shallower depths [45]. As a result, it is possible that the stabilization and subsequent decline in rates of kelp loss from early August to mid-September was caused by a combination of the temperature effect explained above and a gradual decline in urchin displacement and feeding under increasingly high bottom flows as shown by Experiment 2 (see below). The relatively small change in depth (2.6 m) of the front between the onset and end of the field survey, and the strong agreement between observed and expected rates of kelp loss, suggest that temperature had the greatest influence on the urchin front.

Overall, our findings indicate that in habitats with relatively low wave energy, such as CBC, rates of kelp bed destruction by urchin fronts can be predicted from basic knowledge of 1) relationships among individual feeding, temperature, and body size, which we established herein for early summer (June and July) in eastern Newfoundland. These relationships should also be determined for other kelp species and times of the year to incorporate likely temporal variation in urchin-kelp interactions; 2) mean kelp biomass in the bed as well as the number and size structure of urchins at the front, which can be determined accurately by way of a few hours of field work; and 3) sea temperature at the site, which can be obtained for long periods of time from inexpensive and easy-to-install temperature loggers. Continuous records of temperature over months and years should help anticipate times when temperature is likely to reach thermal tipping ranges (12–15°C in the present study) in urchin feeding, which is critical information for marine resource management purposes.

In Nova Scotia (NS), Lauzon-Guay and Scheibling [28] found no statistical relationships between water temperature and the rate of advance of, or urchin density at, a front at temperatures between 0.8 and 17°C. However, the rate of front advance at their site decreased substantially beyond 17°C, with a few instances where the front retreated away from the kelp [28]. The sharp drop in urchin feeding around 15°C and above in our Experiment 1 is consistent with the latter result. Lauzon-Guay and Scheibling [28] also found negative relationships between SWH across the 0.5–2 m range, and urchin movement or density at the front. In another study at the same site, variation in urchin density at the front over 24 d was negatively correlated with SWH across the 0.5–1.5 m range [29]. That in NS temperatures <17°C did not seem to influence urchin-kelp interactions, while the wave environment markedly affected urchin abundance at the front [28, 29], suggests that effects of wave action can override those of temperature when SWH is consistently >0.5 m. In the present study, SWH never exceed 0.51 m, indicating that the wave environment was generally too mild to overcome the influence of temperature. Our study of the relationship between wave velocity and grazing of kelp lines by urchins in a controlled wave environment (Experiment 2) provides the first experimental demonstration of the mechanistic underpinnings for the latter findings.

We found that aggregative feeding rates were: 1) >2.5 times higher in the absence of waves than at intermediate [0.2 m s^-1^] and high [0.3 m s^-1^] wave velocities [no perceptible difference with low, 0.1 m s^-1^, velocity]; and 2) >1.5 times higher in summer [late August to early October] when temperature was within the 12–15°C tipping range of Experiment 1, than spring [April-May] when temperature was lower, ~5°C. These findings, together with significant decreases in both seasons in the proportion of urchins feeding and climbing on the tank walls as wave velocity increased, demonstrate the pervasive effect that wave action can have on feeding and displacement in *S*. *droebachiensis*. That significantly more urchins remained stationary outside of the area swept by kelp in spring than summer suggests that rising temperature may affect specific components of the behavioral repertoire of the green sea urchin. However, this effect was confounded with season in our experiment, which was designed to explore temporal differences as opposed to the strict effect of temperature on these relationships. These results must therefore be interpreted cautiously because factors other than temperature, including reproductive stage of urchins, food availability, and the frequency and intensity of wave storms, change with season and may also affect movement and foraging in *S*. *droebachiensis* [20]. Kawamata [15] documented a similar effect in the urchin *Mesocentrotus nudus* (formerly *Strongylocentrotus nudus*): displacement decreased with increasing wave velocity and ceased at ~0.7 m s^-1^. The latter stopping velocity is more than twice the 0.3 m s^-1^ reported herein and in a study of the ability of *S*. *droebachiensis* to contact the seaweed *Desmarestia viridis* [37]. Altogether, these findings reinforce the notion that urchins are sensitive to changes in the hydrodynamic environment, and that tolerance limits are species-specific.

Our study of the relationships among urchin density, sea temperature, and SWH at CBC yielded foundational results for the largely unstudied region of Newfoundland. Urchin density was affected by variation in sea temperature, but not SWH. Density in the front and within the first 2 m of kelp ahead of the front was positively and negatively correlated to sea temperature, respectively. These results suggest that as sea temperature increases, urchins in the lower bed migrate to the front, therefore increasing the destructive potential of urchins on kelp. That urchin density several meters below the front did not vary with temperature and SWH, further supports the notion that the typical increase in urchin density in fronts during summer [23, 24, 28] is mainly a result of urchins in the bed accumulating in the front as the latter advances through kelp. Further studies are required to test the suggestion that urchins in the lower bed are generally attracted by kelp fragments and associated waterborne chemicals that may settle and diffuse ahead of the plowing front as kelp are being grazed down.

### Conclusion and future research directions

Our integrated approach to the study of individual and aggregative feeding in the green sea urchin, *Strongylocentrotus droebachiensis*, provides the first compelling evidence that water temperature, and not only hydrodynamic forces, can predict kelp bed destruction by urchin fronts in shallow reef communities. By contrast with other systems [28, 29], the hydrodynamic environment at our study site was generally too calm to overcome the effects of temperature on urchin feeding. This finding speaks to the importance of making accurate climate change predictions if we are to anticipate which of the thermal and hydrodynamic environments will be a more important driving force of urchin-kelp dynamics at local and regional scales. The identification of thermal and hydrodynamic thresholds and gradients that trigger shifts in individual and aggregative feeding also has several important conceptual and operational ramifications. Firstly, it highlights the importance of considering thermal regimes in studies of urchin-kelp interactions and kelp-bed boundary dynamics, especially in environments dominated by low hydrodynamic forces where urchins can displace, aggregate, and feed upon kelp more readily. Secondly, it provides novel and vital information, which can feed mathematical models aimed at predicting the timing and magnitude of community phase shifts, with potential applications for the development of a sustainable urchin fishery in Newfoundland [68, 69]. Longer-term experimental and mensurative studies of urchin-kelp interactions at multiple sites spanning broader geographical, thermal, and hydrodynamic ranges are required to test the generality of our findings. Our results support the notion that urchin feeding generally conforms to the basic predictions of the rising MTE. Given 1) the functional importance of urchins in shallow rocky reefs [20]; 2) the influence of sea temperature on their ability to feed [as demonstrated by the present study]; and 3) ongoing global shifts in sea temperature and state induced by climate change [16, 17], studying urchin-seaweed-predator interactions within the conceptual foundations of the MTE holds high potential for improving capacity to predict and manage shifts in marine food web structure and productivity.

## Supporting Information

S1 Table Details of buoys and correlation tests (Pearson’s product-moment correlation) used to examine the fit between mean daily SWH recorded by the water pressure logger at Cape Boone Cove (CBC) and mean daily SWH recorded by surface buoys located offshore (see Materials and methods for a description of SWH calculations). (DOCX)

S2 Table Details of the model parameters from the various statistical analyses presented in this study. (DOCX)

S3 Table Pearson’s product-moment correlation of residuals versus lagged residuals for data in Table 5 and Table 6. (DOCX)
