# Supplementary material for: Thermal and Hydrodynamic Environments Mediate Individual and Aggregative Feeding of a Functionally Important Omnivore in Reef Communities
Source: PLoS One. 2015 Mar 16;10(3):e0118583. doi: 10.1371/journal.pone.0118583 (PMC4361626; doi:10.1371/journal.pone.0118583)
Supplement: S1 Table — (DOCX) [file pone.0118583.s001.docx]

**S1 Table.** **Details of buoys and correlation tests (Pearson’s product-moment correlation) used to examine the fit between mean daily SWH recorded by the water pressure logger at Cape Boone Cove (CBC) and mean daily SWH recorded by surface buoys located offshore (see Materials and methods for a description of SWH calculations).**

| **Buoy or site** | **Location** | **Distance**  **to CBC (km)** | **Data availability** | **Mean (±SE)**  **SWH (m)** | **Sample size (n)** | **Pearson’s *r*** | ***p*** |
| --- | --- | --- | --- | --- | --- | --- | --- |
| SmartAtlantic Placentia Bay^1^ | 46°58’48.6” N  54°41’9.5” W | 150 | 3 Jul - 25 Sep 2012 | 1.568±0.078 | 85 | 0.474 | <0.001 |
| C44139 Banqureau Bank^2^ | 44°16’12.0” N  57°4’48.0” W | 470 | 3 Jul - 25 Sep 2012 | 1.294±0.063 | 85 | 0.502 | <0.001 |
| C44251 Nickerson Bank^2^ | 46°26’24.0” N  53°23’24.0” W | 100 | 3 Jul - 14 Jul 2012 | 1.588±0.143 | 12 | 0.596 | 0.041 |
| CBC | 47°18’30.4” N  52°47’11.1” W | - | 3 Jul - 25 Sep 2012 | 0.316±0.008 | 85 | - | - |

^1^data source: http://www.smartatlantic.ca/PlacentiaBay/buoy.php

^2^data source: http://www.meds-sdmm.dfo-mpo.gc.ca/isdm-gdsi/waves-vagues/search-recherche/index-eng.asp
