# Supplementary material for: Thermal and Hydrodynamic Environments Mediate Individual and Aggregative Feeding of a Functionally Important Omnivore in Reef Communities
Source: PLoS One. 2015 Mar 16;10(3):e0118583. doi: 10.1371/journal.pone.0118583 (PMC4361626; doi:10.1371/journal.pone.0118583)
Supplement: S2 Table — (DOCX) [file pone.0118583.s002.docx]

**S2 Table. Details of the model parameters from the various statistical analyses presented in this study.** Refer to core tables listed in the first column for general results of the statistical analyses.

| **Table 1** | **Range** | **Parameter** | **Coefficient** | **SE** | ***t*-ratio** | ***p*** |
| --- | --- | --- | --- | --- | --- | --- |
|  | [3-12]°C | Intercept | -814.9 | 206.7 | -3.94 | <0.001 |
|  |  | Temp | 36.8 | 14.8 | 2.49 | 0.016 |
|  |  | Size | 31.6 | 4.2 | 7.47 | <0.001 |
|  | ]12-18]°C | Intercept | -2363.9 | 656.3 | -3.60 | 0.001 |
|  |  | Temp | 140.9 | 43.3 | 3.25 | 0.002 |
|  |  | Size | 122.0 | 15.5 | 7.87 | <0.001 |
|  |  | Temp*Size | -7.0 | 1.0 | -6.80 | <0.001 |
|  |  |  |  |  |  |  |
| **Table 2** |  | **Parameter** | **Coefficient** | **SE** | ***t*-ratio** | ***p*** |
|  |  | Intercept | 288.3 | 28.2 | 10.21 | <0.001 |
|  |  | Waves: |  |  |  |  |
|  |  | Null | 199.5 | 48.9 | 4.08 | <0.001 |
|  |  | Low | 73.6 | 48.9 | 1.50 | 0.138 |
|  |  | Intermediate | -99.9 | 48.9 | -2.04 | 0.046 |
|  |  | Season: |  |  |  |  |
|  |  | Spring | -73.6 | 28.2 | -2.60 | 0.012 |
|  |  | Waves*Season: |  |  |  |  |
|  |  | Null*Spring | -15.5 | 48.9 | -0.32 | 0.752 |
|  |  | Low*Spring | -18.9 | 48.9 | -0.39 | 0.701 |
|  |  | Intermediate*Spring | 43.0 | 48.9 | 0.88 | 0.384 |
|  |  |  |  |  |  |  |
| **Table 4** | **Activity or location** | **Parameter** | **Coefficient** | **SE** | ***t*-ratio** | ***p*** |
|  | Feeding | Intercept | -0.65 | 0.05 | -13.38 | <0.001 |
|  |  | Waves: |  |  |  |  |
|  |  | Null | 0.5 | 0.08 | 6.34 | <0.001 |
|  |  | Low | 0.2 | 0.08 | 2.13 | 0.038 |
|  |  | Intermediate | -0.2 | 0.08 | -2.20 | 0.033 |
|  |  | Season: |  |  |  |  |
|  |  | Spring | -0.1 | 0.05 | -1.56 | 0.126 |
|  |  | Waves*Season: |  |  |  |  |
|  |  | Null*Spring | 0.01 | 0.08 | 0.12 | 0.906 |
|  |  | Low*Spring | 0.2 | 0.08 | 1.81 | 0.077 |
|  |  | Intermediate*Spring | -0.1 | 0.08 | -0.93 | 0.355 |
|  | Underneath the kelp | Intercept | -0.6 | 0.03 | -19.72 | <0.001 |
|  | canopy | Waves: |  |  |  |  |
|  |  | Null | -0.1 | 0.05 | -1.64 | 0.108 |
|  |  | Low | -0.04 | 0.05 | -0.75 | 0.457 |
|  |  | Intermediate | 0.1 | 0.05 | 0.98 | 0.334 |
|  |  | Season: |  |  |  |  |
|  |  | Spring | -0.1 | 0.03 | -2.02 | 0.048 |
|  |  | Waves*Season: |  |  |  |  |
|  |  | Null*Spring | 0.01 | 0.05 | 0.28 | 0.780 |
|  |  | Low*Spring | -0.1 | 0.05 | -1.72 | 0.092 |
|  |  | Intermediate*Spring | 0.1 | 0.05 | 2.10 | 0.041 |
|  | On the tiles outside | Intercept | -0.3 | 0.04 | -8.33 | <0.001 |
|  | of the area swept by | Waves: |  |  |  |  |
|  | kelp | Null | -0.7 | 0.07 | -10.48 | <0.001 |
|  |  | Low | -0.2 | 0.07 | -2.40 | 0.020 |
|  |  | Intermediate | 0.3 | 0.07 | 4.50 | <0.001 |
|  |  | Season: |  |  |  |  |
|  |  | Spring | 0.1 | 0.04 | 2.79 | 0.007 |
|  |  | Waves*Season: |  |  |  |  |
|  |  | Null*Spring | 0.03 | 0.07 | 0.38 | 0.707 |
|  |  | Low*Spring | -0.02 | 0.07 | -0.31 | 0.761 |
|  |  | Intermediate*Spring | -0.03 | 0.07 | -0.38 | 0.707 |
|  | On the tank walls | Intercept | -0.7 | 0.04 | -17.77 | <0.001 |
|  |  | Waves: |  |  |  |  |
|  |  | Null | 0.3 | 0.07 | 4.46 | <0.001 |
|  |  | Low | 0.3 | 0.07 | 5.10 | <0.001 |
|  |  | Intermediate | -0.1 | 0.07 | -2.16 | 0.035 |
|  |  | Season: |  |  |  |  |
|  |  | Spring | -0.04 | 0.04 | -0.96 | 0.343 |
|  |  | Waves*Season: |  |  |  |  |
|  |  | Null*Spring | 0.07 | 0.07 | 1.08 | 0.285 |
|  |  | Low*Spring | -0.01 | 0.07 | -0.08 | 0.936 |
|  |  | Intermediate*Spring | -0.08 | 0.07 | -1.23 | 0.225 |
|  |  |  |  |  |  |  |
| **Table 5** |  | **Parameter** | **Coefficient** | **SE** | ***t*-ratio** | ***p*** |
|  |  | Intercept | 115.5 | 39.3 | 2.936 | 0.012 |
|  |  | SWH | -51.2 | 87.5 | -0.585 | 0.569 |
|  |  | Temp | -4.1 | 2.0 | -1.994 | 0.069 |
|  |  | Zone: |  |  |  |  |
|  |  | Barrens | -116.5 | 55.6 | -2.095 | 0.058 |
|  |  | Front | -62.1 | 55.6 | -1.116 | 0.286 |
|  |  | Kelp Bed | -41.0 | 55.6 | -0.737 | 0.475 |
|  |  | SWH*Zone: |  |  |  |  |
|  |  | SWH*Barrens | 253.1 | 123.7 | 2.046 | 0.063 |
|  |  | SWH*Front | 46.9 | 123.7 | 0.379 | 0.711 |
|  |  | SWH*Kelp Bed | 46.5 | 123.7 | 0.376 | 0.713 |
|  |  | Temp*Zone: |  |  |  |  |
|  |  | Temp*Barrens | 6.3 | 2.9 | 2.171 | 0.051 |
|  |  | Temp*Front | 10.6 | 2.9 | 3.678 | 0.003 |
|  |  | Temp*Kelp Bed | 1.1 | 2.9 | 0.369 | 0.718 |
|  |  |  |  |  |  |  |
| **Table 6** | **Zone** | **Parameter** | **Coefficient** | **SE** | ***t*-ratio** | ***p*** |
|  | Barrens | Intercept | 78.6 | 25.9 | 3.03 | 0.039 |
|  |  | Temp | -0.1 | 2.5 | -0.03 | 0.981 |
|  | Pre-front | Intercept | 95.3 | 23.5 | 4.06 | 0.015 |
|  |  | Temp | -3.5 | 2.2 | -1.57 | 0.192 |
|  | Front | Intercept | 51.7 | 17.4 | 2.97 | 0.041 |
|  |  | Temp | 6.6 | 1.7 | 3.99 | 0.016 |
|  | Bed | Intercept | 72.7 | 6.7 | 10.9 | <0.001 |
|  |  | Temp | -3.0 | 0.6 | -4.66 | 0.010 |
|  |  |  |  |  |  |  |
| **Table 7** | **Data** | **Parameter** | **Coefficient** | **SE** | ***t*-ratio** | ***p*** |
|  | 25 Sep in | Intercept | -374.1 | 473.0 | -0.79 | 0.473 |
|  |  | Slope (Expected) | 9.9 | 7.9 | 1.25 | 0.279 |
|  | 25 Sep out | Intercept | -361.2 | 96.3 | -3.75 | 0.033 |
|  |  | Slope (Expected) | 8.8 | 1.6 | 5.46 | 0.012 |
