# Supplementary material for: Thermal and Hydrodynamic Environments Mediate Individual and Aggregative Feeding of a Functionally Important Omnivore in Reef Communities
Source: PLoS One. 2015 Mar 16;10(3):e0118583. doi: 10.1371/journal.pone.0118583 (PMC4361626; doi:10.1371/journal.pone.0118583)
Supplement: S3 Table — (DOCX) [file pone.0118583.s003.docx]

| **Zone** | ***r*** | ***p*** |
| --- | --- | --- |
| Full analysis (all zones pooled) | -0.408 | 0.053 |
| Barrens | 0.848 | 0.070 |
| Pre-front | -0.630 | 0.255 |
| Front | -0.758 | 0.137 |
| Kelp bed | -0.185 | 0.766 |

**S3 Table. Pearson’s product-moment correlation of residuals versus lagged residuals for data in Table 5 and Table 6.**
